# Combined inhibition of histone deacetylase and cytidine deaminase improves epigenetic potency of decitabine in colorectal adenocarcinomas

**DOI:** 10.1186/s13148-023-01500-1

**Published:** 2023-05-19

**Authors:** Zijiao Tang, Lu Liu, Jürgen Borlak

**Affiliations:** Hannover Medical School, Centre for Pharmacology and Toxicology, Carl-Neuberg-Str.1, 30625 Hannover, Germany

**Keywords:** Colon adenocarcinoma (COAD), Decitabine, Phenylbutyrate acid (PBA), Genome-wide gene and micro-RNA expression, Chromosome distribution, Tumor suppressors, Tetrahydrouridine (THU), Cytidine deaminase (CDA), Gene knockdown validation studies, Clinical translation

## Abstract

**Background:**

Targeting the epigenome of cancerous diseases represents an innovative approach, and the DNA methylation inhibitor decitabine is recommended for the treatment of hematological malignancies. Although epigenetic alterations are also common to solid tumors, the therapeutic efficacy of decitabine in colorectal adenocarcinomas (COAD) is unfavorable. Current research focuses on an identification of combination therapies either with chemotherapeutics or checkpoint inhibitors in modulating the tumor microenvironment. Here we report a series of molecular investigations to evaluate potency of decitabine, the histone deacetylase inhibitor PBA and the cytidine deaminase (CDA) inhibitor tetrahydrouridine (THU) in patient derived functional and p53 null colon cancer cell lines (CCCL). We focused on the inhibition of cell proliferation, the recovery of tumor suppressors and programmed cell death, and established clinical relevance by evaluating drug responsive genes among 270 COAD patients. Furthermore, we evaluated treatment responses based on CpG island density.

**Results:**

Decitabine caused marked repression of the DNMT1 protein. Conversely, PBA treatment of CCCL recovered acetylation of histone 3 lysine residues, and this enabled an open chromatin state. Unlike single decitabine treatment, the combined decitabine/PBA treatment caused > 95% inhibition of cell proliferation, prevented cell cycle progression especially in the S and G2-phase and induced programmed cell death. Decitabine and PBA differed in their ability to facilitate re-expression of genes localized on different chromosomes, and the combined decitabine/PBA treatment was most effective in the re-expression of 40 tumor suppressors and 13 genes typically silenced in cancer-associated genomic regions of COAD patients. Furthermore, this treatment repressed expression of 11 survival (anti-apoptotic) genes and augmented expression of X-chromosome inactivated genes, especially the lncRNA Xist to facilitate p53-mediated apoptosis. Pharmacological inhibition of CDA by THU or its gene knockdown prevented decitabine inactivation. Strikingly, PBA treatment recovered the expression of the decitabine drug-uptake transporter SLC15A1, thus enabling high tumor drug-loads. Finally, for 26 drug responsive genes we demonstrated improved survival in COAD patients.

**Conclusion:**

The combined decitabine/PBA/THU drug treatment improved drug potency considerably, and given their existing regulatory approval, our findings merit prospective clinical trials for the triple combination in COAD patients.

**Supplementary Information:**

The online version contains supplementary material available at 10.1186/s13148-023-01500-1.

## Introduction

According to the global cancer statistics of the WHO, colorectal cancer (CRC) ranks as the third leading cause of cancer-related deaths worldwide with more than 1.8 million new cases and 577.192 deaths in 2020 [1]. Similar incidences are reported for European countries [2]. Notwithstanding, the trends in CRC statistics show a remarkable decline in incidence and mortality among individuals aged > 50 years and are the result of effective screening programs. However, the contrary is seen among patients aged < 50 years [3]. Typically young people are not screened for CRC unless their medical history or genetic risk constellations impose a higher risk for CRC. For instance, lynch syndrome patients are carriers of variant DNA mismatch repair genes and/or deletions of the EpCAM gene and are at higher risk of developing CRC. Evidently, such patients benefit from colonoscopic surveillance, and early detection of CRC is associated with excellent survival [4].

The mainstay of CRC treatment is surgery, especially at early stages of disease, and depending on the clinical stages, it may involve adjuvant chemo- and radiotherapy. To this date, drug treatment for unresectable colorectal cancer is based on 5-fluorouracil (5-FU) chemotherapy in addition to molecular therapies targeting the epidermal growth factor receptor (EGFR) as well as vascular endothelial growth factor (VEGF) with antibodies like bevacizumab. Recently, the combined use of encorafenib, i.e., a BRAF kinase inhibitor, and cetuximab, i.e., an EGFR inhibitory antibody, was approved for the treatment of metastatic colorectal cancer.

Additionally, a therapy with checkpoint inhibitors has been approved by the FDA (pembrolizumab and nivolumab, i.e., PD-1 inhibitor) which is recommended for patients with high microsatellite instability (MSI-H) and deficient mismatch repair (dMMR) [5]. However, immune therapy is not recommended for patients with proficient pMMR/microsatellite stable disease [6], and in a recent ASCO educational book, the subject as to where we stand with immunotherapy in CRC has been addressed [6].

Apart from the above-mentioned therapeutic options, epigenetic therapies hold promise for an improved anticancer treatment with less side effects, and they are most effective in liquid tumors, especially acute myeloid leukemia (AML) and the myelodysplastic syndrome (MDS) [7, 8]. It is somewhat perplexing that epigenetic drug treatment is less promising in the treatment of solid tumors.

In general, epigenetics refers to the state of chromatin and its remodeling and includes the reversible DNA methylation and histone acetylation. Multiple studies report aberrant epigenetic regulation of genes in cancers [9], and the importance of epigenetics in tumorigenesis and metastatic spread of tumor cells is well recognized [10, 11]. Although DNA methylation is a natural occurring modification of DNA and refers to an addition of a methyl group to the 5′ position of the cytosine ring of the so-called CpG islands, such epigenetic control fails in tumors through hypermethylation of promoters of, for instance, tumor-suppressor genes [12].

DNA methyltransferases (DNMTs) are key enzymes in the maintenance of methylation pattern of the genome [13], and drugs that cause epigenetic reprogramming of tumor cells represent an interesting approach to treat cancers. 5-Aza-2′-deoxycytidine (decitabine or DAC) belongs to a group of nucleoside analogues. The drug inhibits the activity of DNMTs and impairs hypermethylation of genes. Specifically, upon cellular uptake, the drug is phosphorylated to the active nucleotide by deoxycytidine kinase, and 5-aza-deoxycytidine is incorporated into DNA of proliferating cells. 5-Aza-deoxycytidine substitutes for the pyrimidine base cytosine, and the 5-azadeoxycytosine-guanine dinucleotide is a substrate for DNMT. The methylation reaction is initiated through nucleophilic attack [14, 15], and this results in a covalent bond of DNMT with cytosine. Typically, the reaction is reversed by ß-elimination. However, in the case of 5-aza-deoxycytidine, the ß-elimination is blocked and the DNMT enzyme is trapped. Consequently, DNMT is unable to transfer the methyl group from S-adenosylmethionine (SAM), and this results in “hypomethylation” of CpG islets. Moreover, at higher concentrations DAC becomes cytotoxic to abnormal hematopoietic cells. First approved in 2006, the drug is nowadays used to treat all types of the myelodysplastic syndrome [16].

Although highly effective in some liquid tumors, its therapeutic failures in solid tumors remain unclear. Nonetheless, its rapid deamination by cytidine deaminase leads to short half-live of decitabine of about 30 min and its pharmacological inactivation [17]. In fact, cytidine deaminase (CDA) converts cytidine to uracil and is a major salvage pathway for pyrimidine nucleotides. To overcome current limitations, prodrugs of decitabine, which are resistant to deamination, were reported to improve therapeutic efficacy in animal models [18].

Moreover, there is clear evidence from preclinical studies for tetrahydrouridine (THU) to increase bioavailability of decitabine [19]. Specifically, through nucleophilic attack of the water/zinc complex, CDA catalyzes the conversion of cytidine to uridine in the pyrimidine biosynthetic pathway. However, THU binds to zinc instead of the water molecule of the C4 hydroxyl moiety of the pyrimidic ring, and this blocks the CDA activity [20].

Furthermore, histone acetylation is of critical importance for an open state chromatin. However, tumor cells instruct histone deacetylases to condense chromatin, thereby preventing gene transcription [21]. Inhibitors of histone deacetylases (HDACi) allow for an open chromatin state, and 4-phenylbutyric acid (PBA), i.e., an aromatic fatty acid, inhibits histone deacetylases. Molecular docking studies revealed PBA’s mode of action. It binds to the Zn^2+^ coordination system of the active-site pocket of histone deacetylases, thereby blocking its catalytic activity [22]. PBA is effective against class 1 and 2 histone deacetylase. Additionally, it functions as a chaperone in the unfolded protein response, in ER stress and changes mitochondrial metabolism [23]. The FDA approved PBA for the treatment of urea cycle disorders [24].

Being effective at the lower millimolar range, PBA displays low toxicity and high lipophilicity, which makes it an interesting drug for the combined use with decitabine. Indeed, research on various metastatic melanoma cell lines demonstrated effectiveness in the inhibition of cell proliferation, and the combined drug treatment recovered the expression of the tumor-suppressor 14-3-3σ [25].

The use of epigenetic drugs with other drugs such as etoposide, doxorubicin and bortezomib improves therapeutic potency in assays with various cancer cell lines [26], and Belinky et al. demonstrated improved efficacy in lung cancer following treatment of mice with a combination of decitabine and PBA [27]. Likewise, the combined use of decitabine with an anti-CD105 therapeutic antibody caused a more durable anti-leukemic effect in a xenograft model of acute myeloid leukemia [28], and Venetoclax, i.e., an oral inhibitor of BCL-2 combined with decitabine or azacitidine, achieved nearly 70% complete remission in treatment-naive, elderly patients with acute myeloid leukemia [29].

Moreover, the combined use of cisplatin and decitabine improved therapeutic efficacy in a HCC xenograft disease model [30], and low-dose decitabine treatment enhanced the effect of PD-1 blockade in colorectal cancer with microsatellite stability by re-modulating the tumor microenvironment [31]. In a recent review on the epigenetics of colorectal cancer, the therapeutic potential of DNA methyltransferases and histone deacetylase inhibitors has been summarized [32].

In regard to genomic responses, and to the best of our knowledge, there is only one study, which investigated the chromatin signatures of microRNAs in 3 different colorectal cancer cell lines following single or combined 5-Aza and PBA treatment [33]. The study offered insight into how epigenetic drugs recovered the function of dysregulated miRNAs in cancer. However, the target genes of miRNAs were not investigated. Additionally, we identified three genomic studies, which either focused on the genomic responses of melanoma cells following treatment with decitabine and/or trichostatin [34] or gastric cancer cells following exposure to decitabine and PBA [35]. Lastly, one study investigated the effects of decitabine and/or trichostatin in the HCT116 and SW480 colon cancer cell lines, and some target genes were validated in colorectal cancer patient samples [36]. Essentially the study highlighted three different methylation epigenotypes, some of which are associated with poor prognosis.

To improve potency, we investigated the effects of single or combined treatment of decitabine and PBA for the rescue of tumor suppressors, cell cycle regulators and cell death/apoptosis-related genes. We used the genomic data as a surrogate endpoint to infer therapeutic efficacy by evaluating pharmacological activity of single or combined treatment in different cancer cell lines. We assessed the effects of decitabine on the regulation of DNMT1, cell cycle, cell proliferation and programmed cell death, and compared the drug treatment effects in p53 null and wild-type human colon cell lines and imaged the growth of EdU-labeled colon cancer cells by fluorescence microscopy. In addition, the effects of decitabine and PBA on the regulation of microRNAs and their cross-talk with target genes have not been reported, even though miRNAs are of critical importance in the control of gene transcription. Therefore, we constructed regulatory gene networks by considering the interplay of miRNA and target genes. Moreover, we identified the chromosomal distribution of drug-responsive genes and evaluated CpG islands density of promoters of regulated genes. We also assessed H3 acetylation pattern after PBA and the combined PBA/DAC treatment by considering 5 different lysine residues of histone H3 and investigated pharmacological inhibition of CDA by THU. We show THU to improve the potency of DCA considerably as evidenced by the significant increase in the inhibition of cell proliferation. Finally, we assessed the clinical relevance of drug response genes in Kaplan–Meier survival plots of 270 colon cancer patients.

Overall, our study aimed at identifying drug response genes as a molecular rationale for epigenetic drug treatment, and we demonstrate improved potency of DAC by blocking its rapid deamination and by co-administration with the histone deacetylase inhibitor PBA. Together, our study provides a rationale for the combined use of DCA, PBA and THU in colorectal cancer patients and warrants clinical trials.

## Results

### Inhibition of cell proliferation after decitabine or PBA treatment

**Fig. 1:**
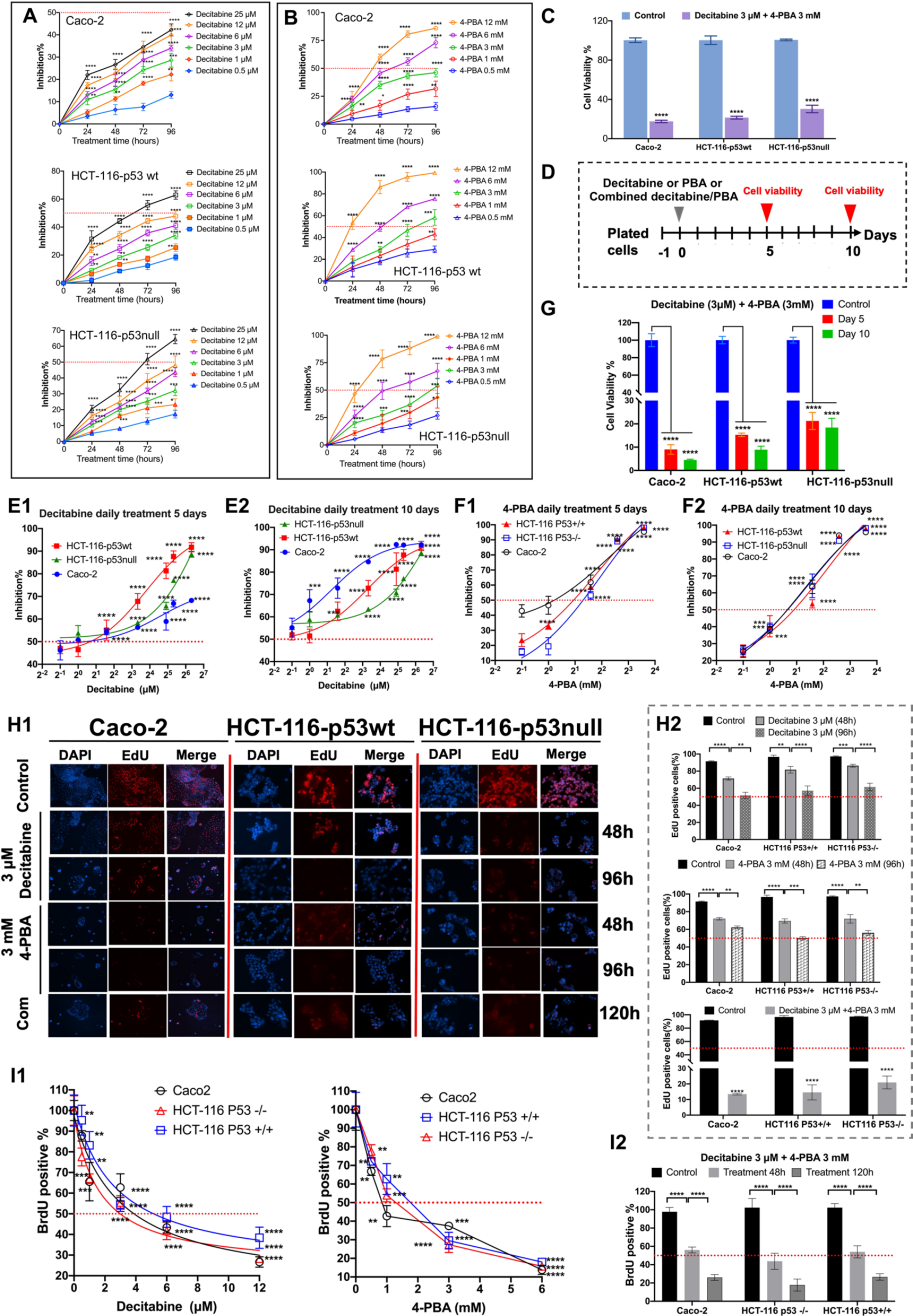
The effects of decitabine, PBA and the combined treatment on cell viability and proliferation. **A** Inhibition of cell proliferation of Caco-2, HCT-116-p53wt and HCT-116-p53null cells following single decitabine treatment at different drug concentration for up to 96 h. **B** Inhibition of cell proliferation of Caco-2, HCT-116-p53wt and HCT-116-p53null cells following single PBA treatment at different drug concentrations for up to 96 h. **C** Cell viability of Caco-2, HCT-116-p53wt and HCT-116-p53null cells following the combined decitabine (3 µM) and PBA (3 mM) single treatment for 120 h. **D** Treatment scheme of Caco-2, HCT-116-p53wt and HCT-116-p53null cells following daily treatment with decitabine or PBA or combined decitabine/PBA treatment for 5 and 10 days. **E** Inhibition of cell proliferation of Caco-2, HCT-116-p53wt and HCT-116-p53null cells following daily decitabine treatment at different drug concentrations for 5 days (**E1**) and 10 days (**E2**). **F** Inhibition of cell proliferation of Caco-2, HCT-116-p53wt and HCT-116-p53null cells following daily PBA treatment at different drug concentrations for 5 days (**F1**) and 10 days (**F2**). **G** Cell viability of Caco-2, HCT-116-p53wt and HCT-116-p53null cells following combined decitabine and PBA treatment for 5 days and 10 days. **H** Fluorescent phase contrast imaging of Edu-labeled Caco-2 cells following daily decitabine, PBA and the combined drug treatment for 48 h and 96 h (**H1**). The histograms show the percentage change of Edu-positive cells (**H2**). The image analysis is based on three independent experiments. Each microscopic field of view is divided into four quadrants, and a total of 12 images per epi-drug treatment were evaluated. All images are at 20X- magnifications. **I** BrdU labeling of colon cancer cells following decitabine or PBA daily treatments for 96 h (**I1**) and the combined decitabine/ PBA daily treatment for 48 h and 120 h (**I2**). We observed a dose proportional inhibition of cell proliferation following drug treatment. Statistical significance *p < 0.05, **p < 0.01, ***p < 0.001, ****p < 0.0001

We performed dose range-finding studies with decitabine (Fig. 1A) or PBA (Fig. 1B) after single drug treatment of colon cancer cells for up to 96 h. The results of the MTT assay clearly demonstrated a time- and concentration-dependent inhibition of tumor cell viability, which ranged between 42% (Caco-2) and 65% (HCT-116 cell lines), at the highest drug concentration. Furthermore, the inhibition of cell proliferation between p53wt and p53 null colon cancer cells was similar, i.e., 62.8% and 64.5% (Fig. 1A). Notwithstanding, single PBA treatment was more effective in inhibiting cell proliferation and depending on the cell line ranged between 85 (Caco-2)—99% (HCT-116 cell lines) at the highest PBA concentration. Once again, inhibition of cell proliferation was similar between the HCT-116p-53wt and the p53null cell line, i.e., 99 and 98%, respectively.

Subsequently, we investigated the effects of the combined decitabine and PBA treatment at clinically relevant therapeutic drug concentrations and observed highly significant inhibition of cell proliferation, i.e., 82.4%, 78.5% and 69.7%, respectively, in the Caco-2, HCT-116-p53wt and HCT-116-p53null cell lines (Fig. 1C). Therefore, the combined drug treatment is more effective at clinically relevant concentrations when compared to single drug treatment and resulted in almost complete inhibition of cell growth (p < 0.001, Additional file 1: Table S1).

We also investigated the effects of different drug concentrations following single drug (decitabine or PBA) or combined (Fig. 1D) daily drug treatments for 5 or 10 days. With Caco-2 cells, prolonged treatments sensitized the cell line to decitabine. At the highest drug concentration, the inhibition of cell proliferation increased from 68 to 92% (Figs. 1E1-2), while at clinically relevant concentrations of 3 µM (Cmax in patients) the change was 54.3–68.5%. It appears that the HCT cell lines are more sensitive to decitabine. After daily decitabine treatment for 10 days, the inhibition of cell proliferation increased from 55.1 to 62.5% and 55.81 to 60.8%, respectively, in the HCT-116-p53wt and HCT-116-p53null cell lines at 3 µM drug concentration. For PBA treatments (Fig. 1F), we noted a strict dose dependency especially after daily treatment for 10 days. Here the dose–response curves are almost superimposable for the different colon cancer cell lines (Fig. 1F2). However, at lower drug concentrations, the cell lines differed in their sensitivity to PBA after daily dosing for 5 days (Fig. 1F1).

**Fig. 2:**
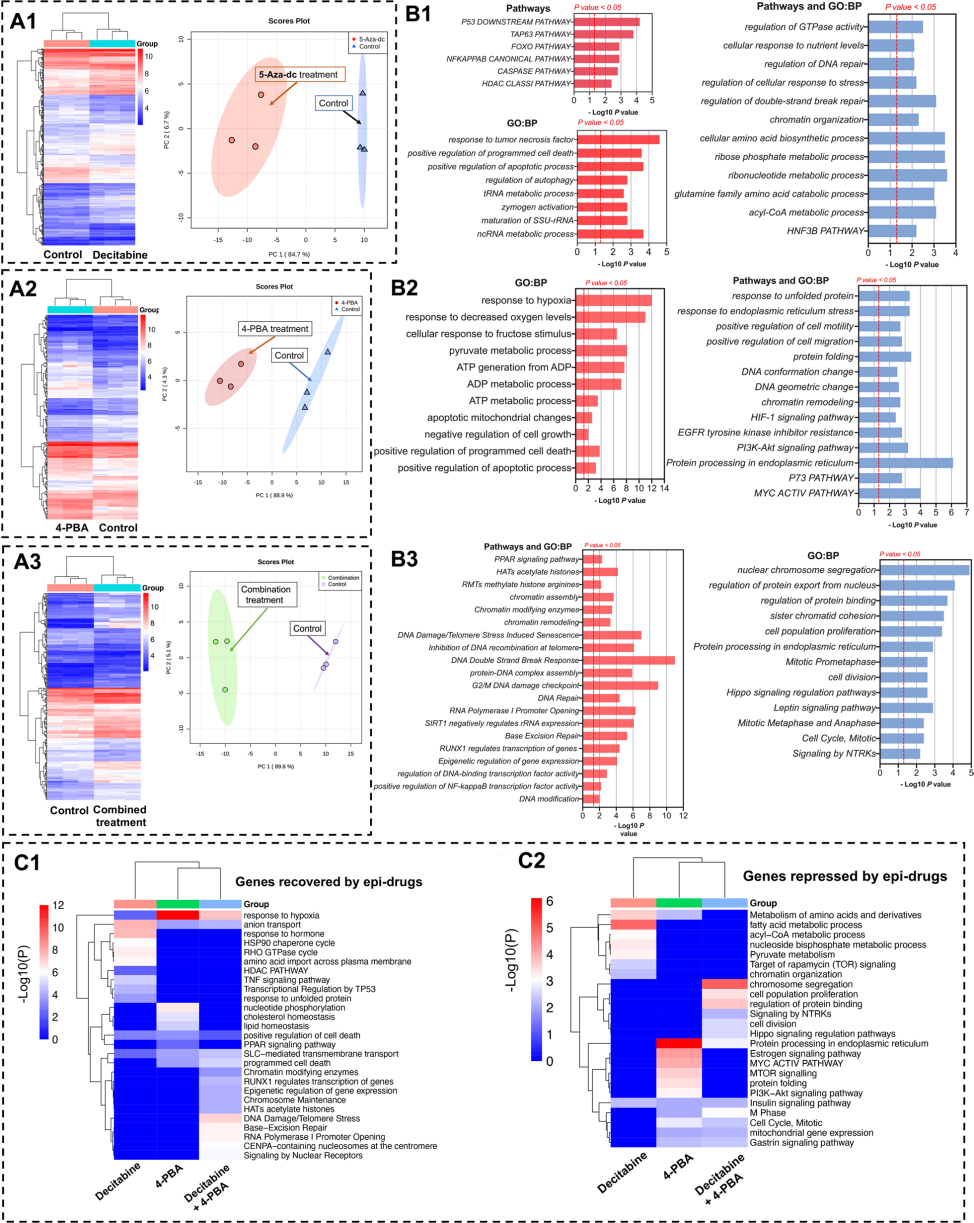
Genomic profiling and pathway enrichment analysis following decitabine, PBA and combined drug treatment of Caco-2 cells for 96 h. **A** Heat maps and principal component analysis (PCA) of genomic data after decitabine (**A1**), PBA (**A2**) and the combined drug treatment (**A3**) of Caco-2 cells. The data are compared to DMSO vehicle control treatment. **B** Metascape gene ontology terms and enriched pathways for up- and downregulated genes in response to decitabine (**B1**), PBA (**B2**) and the combined drug treatment (**B3**). Red colored histograms refer to enriched GO terms and pathways of upregulated genes. The blue colored histograms refer to enriched GO terms and pathways of downregulated genes. **C** A comparison of significantly enriched ontology terms and pathways associated with upregulated (**C1**) and downregulated genes (**C2**) in response to decitabine, PBA and the combined treatment of Caco-2 cells

Next, we considered the effects of combined decitabine/PBA daily treatments at clinically relevant drug concentrations (Fig. 1G). Once again, we observed a highly significant reduction in cell proliferation to 4.6%, 8.9% and 19.5%, respectively, for the Caco-2, HCT-116-p53wt and HCT-116-p53null cell lines following 10 days of treatment. The data imply that repeated daily treatments are more effective in the inhibition of growth (p < 0.001) when compared to single treatments (see Fig. 1C and G, Additional file 1: Table S1).

### Fluorescence microscopy imaging of EdU-labeled colon cancer cell lines and quantification of BrdU DNA labeling

We evaluated cell proliferation and DNA synthesis by fluorescence microscopy of EdU (5-ethynyl-2´-deoxyuridine)-labeled Caco-2, HCT-116-p53wt and HCT-116-p53null cells. The assay enabled direct measurement of DNA synthesis, and we treated the cell cultures at clinically relevant drug concentrations with decitabine, PBA or the combination for 48 h, 96 h and 120 h. We labeled the cell cultures with the EdU dye according to the manufacturer’s recommendations and used the Hoechst 33,342 dye as a nuclear counterstain. We show representative images in Fig. 1I1 and determined the number of EdU-positive cells in relation to the Hoechst 33,342 nuclear counterstain. Typically, we evaluated the images of three independent experiments by dividing representative images (n = 12) into four quadrants. First, we determined the number of Hoechst 33,342 positive cells. Second, we selected the fluorescent filter cube TxRed and determined the number of EdU-labeled cells for the same image. Third, we calculated the percentage of EdU-labeled cells and reported the results in Fig. 1I2. Following daily treatments for 96 h, decitabine treatment inhibited cell proliferation up to 50% among the three different cancer cell lines. Likewise, treatment of the cancer cell lines with 3 mM PBA inhibited cell proliferation by 50%. Nonetheless, the HCT-116-p53wt cell line is more sensitive to the treatment effects (p < 0.02) when compared to the other two cell lines (Additional file 1: Table S1). Strikingly, the combined decitabine/PBA treatment was highly effective and blocked cell proliferation by 80–90%; Caco-2 cells are most sensitive to this treatment when compared to the other two cell lines (p < 0.03).

Additionally, we performed BrdU labeling studies with decitabine or PBA for up to 96 h (Fig. 1J1) and investigated the effects of the combined drug treatment for up to 120 h (Fig. 1J2). The results for the 48-h treatment are given in Additional file 2: Fig. S1B. Essentially, the dose–response curves for the three different cell lines are comparable. However, at clinically relevant 3 µM decitabine concentration, the HCT-116-p53wt cell line is more sensitive to the drug treatment when compared to the other two cell lines (p < 0.037). PBA produced similar results, and at the 3 mM PBA concentration, both HCT-116 cell lines were more sensitive to the drug treatment when compared to the Caco-2 cell line (p < 0.035). Once again, the combined decitabine/PBA treatment is superior to the single drug treatment (p < 0.001, Additional file 1: Table S1) in inhibiting cell proliferation, and we observed a time-dependent increase in the sensitivity of the colon cancer cell lines to the combined decitabine/PBA treatment (Fig. 1J2). Therefore, daily treatment sensitized the colon cancer cells to the epigenetic drug treatment and even more remarkably, the p53null HCT-116 cancer cell line was most sensitive to the combined drug treatment. Here, the number of BrdU-positive labeled cells declined to 17.7% as compared to 26.1% and 26.5%, respectively, for the Caco-2 and HCT-116p53wt (p < 0.0190).

### EC/IC50 estimation

Based on the MTT assay, we determined the EC50 values for decitabine and PBA following daily treatments of the cancer cell lines for 5 and 10 days (Additional file 1: Table S1; Additional file 2: Fig. S1). The Caco-2 cell line is most sensitive to decitabine treatments with an EC50 of 0.78 µM and 0.43 µM, respectively, following treatments for 5 and 10 days. The difference in EC50 values is statistically significant (p < 0.001). Likewise, we computed EC50 values of 3.24 µM and 5.70 µM for the HCT-116-p53wt and HCT-116-p53null cell lines, and with the p53 null cell line, daily treatments for 10 days were more effective when compared to the 5 day treatments, i.e., 5.68 µM versus 3.68 µM (p < 0.001, Additional file 2: Fig. S1). Together, the sensitivity of the colon cancer cell lines follows the order Caco-2 < HCT-116-p53wt < HCT-116-p53null cells, and the differences in EC50 values are statistically significant (p < 0.001, Additional file 1: Table S1).

The sensitivity of the cancer cell lines to PBA treatment is similar to that of decitabine, and the EC50 values are 1.78, 2.46 and 3.1 mM, respectively, for the Caco-2, HCT-116-p53wt and HCT-116-p53null cell line. Unlike decitabine, however, the EC50 values for PBA did not differ after 5 or 10 days of daily treatments.

### Genomic profiling of Caco-2 cells after decitabine, 4-PBA and the combined treatment

We performed genome-wide expression profiling to identify differentially expressed genes (DEGs) and miRNAs (DEMs) after decitabine, 4-PBA and the combined drug treatment.

Based on FDR-adjusted p-values and a threshold of FC > 1.5, we identified 537 DEGs and 317 DEMs following daily decitabine treatment for 96 h (Fig. 2A1, Additional file 3: Table S2). The heat map and the principle component analysis (PCA) clearly segregate the treatment groups from the controls (Fig. 2A), and decitabine treatment caused an upregulation of 291 genes (54.2% of DEGs). For the upregulated genes, the Metascape gene annotations list p53-mediated apoptosis, upregulation of tumor suppressors and autophagy as highly enriched terms. Among the 246 downregulated genes (45.8% of DEGs), enriched terms emphasize the regulation of DNA repair, chromatin organization and FOXOA2 (HNF3ß) pathways (Fig. 2A1 and B1).

Treatment of Caco-2 cell cultures with the histone deacetylase inhibitor PBA defined 224 DEGs of which 133 were up- (59.3%) and 91 (40.7%) downregulated. Once again, the heat map and the PCA clearly segregated the controls from the treatment groups (Fig. 2A2), and based on Metascape annotations of upregulated genes, response to hypoxia, apoptotic mitochondrial changes, negative regulation of cell growth and positive regulation of programmed cell death are enriched terms. Importantly, PBA treatment selectively induced the expression of the drug transporter SLC15A1/PEPT1, and this peptide transporter is of critical importance for the cellular uptake of decitabine (nearly threefold upregulation, Additional file 3: Table S2). Its epigenetic activation by PBA implies a significant role of HDAC1 in the regulation of SLC15A1/PEPT1. Indeed, re-expression of epigenetically silenced SLC15A1/PEPT1 sensitized colorectal cancer cells for drug treatment as recently demonstrated [40].

PBA treatment also caused the repression of gene expression and based on Metascape annotations enriched terms are: Response to ER stress, positive regulation of cell motility and migration, DNA conformational changes, chromatin remodeling, EGFR tyrosine kinase inhibitor resistance and MYC activity pathways.

Moreover, the combined daily treatment of Caco-2 cells with decitabine and PBA resulted in 230 DEGs of which 147 (63.9%) were up- and 83 (36.1%) were downregulated. As shown in Fig. 2A3, the controls are segregated from the decitabine/PBA combined treatment, and we employed the following treatment protocol: Initially, we treated the Caco-2 cells with decitabine/PBA for 72 h followed by singular PBA treatment for another 2 days. Among enriched terms of upregulated genes are histone acetylation, chromatin-modifying enzymes, DNA double-strand brake response and DNA damage/telomere stress-induced senescence and G2/M DNA damage checkpoint (Fig. 2B3). Once again, the combined drug treatment resulted in induced expression of the SLC15A1/PEPT1 transporter; however, decitabine alone did not influence the expression of this transporter in the Caco-2 colon cancer cell line, and this reinforces the notion that its epigenetic regulation is primarily driven by HDAC1 (see below for mechanistic studies).

Vice versa, for downregulated genes, enriched GO terms are nuclear chromosome segregation, sister chromatid cohesion, cell proliferation, cell division, mitotic metaphase and anaphase and signaling by NTRK tyrosine-receptor-kinase.

### Comparative genomic analysis reveals distinct pathways induced by epigenetic drugs

Depicted in Fig. 2C are heat maps which highlight significantly enriched ontology terms after treatment of the Caco-2 cell line with decitabine, PBA or the drug combination. Some terms are common between the DNA methylation and histone deacetylase inhibitor; however, there are also important differences. In the case of upregulated genes (Fig. 2C1), pathways specifically linked to decitabine treatment are response to hormone, HSP90 chaperon cycle, Rho GTPase cycle and amino acid import across plasma membrane, TNFα and p53 signaling. Conversely, pathways specifically linked to PBA treatments are nucleotide phosphorylation, cholesterol and lipid homeostasis. For the combined drug treatment, chromatin-modifying enzymes, epigenetic gene regulation, histone acetylation, regulation of the centromere-specific histone centromeric protein A (CENP-A) nucleosome and RNA polymerase I promoter opening are significantly enriched terms. Evidently, the combined drug treatment caused marked changes in the epigenetic regulation of gene transcription, and we discuss further examples below.

We also considered enriched pathways specifically associated with downregulated genes (Fig. 2C2). DAC treatment caused repression of amino- and fatty acid metabolism. Conversely, PBA treatment repressed MYC and PI3k-AKT signaling pathways, and the combined decitabine/PBA treatment repressed chromatin segregation and cell proliferation, protein processing in endoplasmic reticulum and HIPPO signaling pathways. Finally, PBA and the combined drug treatment repressed mitotic cell cycle, M-phase genes and mitochondrial gene expression. Notwithstanding and common to all drug treatments is the repression of insulin signaling pathways.

### Decitabine and PBA treatment of colon cancer cells recover expression of apoptosis genes

**Fig. 3:**
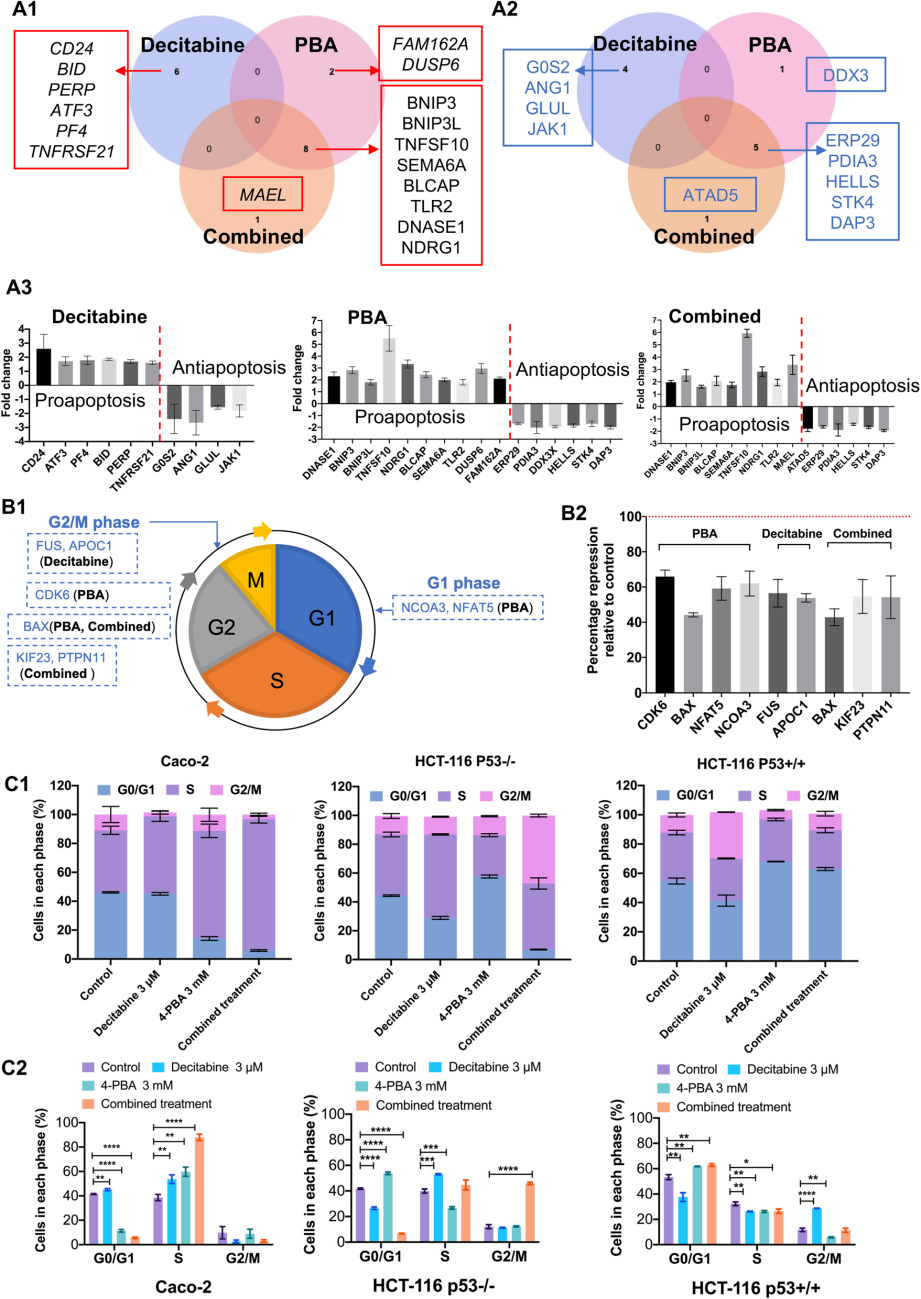
Regulation of apoptosis and cell cycle-related genes and cell cycle analysis in colon cancer cells following decitabine, PBA and the combined drug treatment. **A** Depicted is a Venn diagram highlighting unique and common apoptosis-related gene regulations following decitabine, PBA and the combined drug treatment. Together, we identified 17 upregulated pro-apoptosis (**A1**) and 11 repressed anti-apoptosis regulated genes (**A2**). The histogram highlights the gene expression changes after different epi-drug treatments (**A3**). **B** Cell cycle-related gene regulations following daily decitabine, PBA and the combined drug treatment for 96 h. Drug treatment repressed 8 cell cycle genes with functions in G1 and G2/M phases (**B1**), respectively. The histogram shows the percentage repression relative to the DMSO controls (**B2**). **C** Cell cycle analysis of the 3 different colon cancer cell lines following decitabine, PBA and the combined daily treatment for 72 h. We performed FACS assays with the Dye Cycle Violet stain. The data are percentage cells in the different phases of the cell cycle (**C1**). The statistical analysis is based on an unpaired t-test of three independent experiments (**C2**). Drug treatment caused inhibition of cell cycle progression in different phases of the cell cycle

We observed 28 apoptosis-related genes which are regulated following DAC, 4-PBA and the combined drug treatment of Caco-2 cells. Specifically drug treatment caused induced expression of 17 pro-apoptotic genes and repression of 11 anti-apoptotic genes. The Venn diagram shown in Fig. 3A highlights the drug-specific regulations for induced pro-apoptotic (Fig. 3A1) and repression of anti-apoptotic genes (Fig. 3A2), while Fig. 3A3 informs on individual gene expression changes. Specifically, decitabine treatment caused upregulation of six apoptosis genes, whereas two genes were uniquely upregulated by PBA, i.e., FAM162A and DUSP6, and the combined drug treatment induced specifically the expression of MAEL (Maelstrom Spermatogenic Transposon Silencer). Note this protein is a regulator of the intrinsic apoptotic signaling pathway following DNA damage. Furthermore, there are eight genes commonly regulated whose expression is induced by PBA or the combined decitabine/PBA treatment.

In Additional file 4: Table S3, we compile a list of genes involved in the apoptotic pathway and provide experimental evidence of drug treatment induced apoptosis, i.e., PARP cleavage and the regulation of various caspases further below. Specifically, DAC treatment induced CD24 expression, and this GPI-anchored protein promotes DNA damage-induced apoptosis via NFκB signaling [41]. We also observed induced BID, i.e., a member of the BCL-2 family of cell death regulators following decitabine treatment, and independent research demonstrated BID cleavage on the mitochondria to be essential for caspase-8-induced cytochrome c release [42]. Likewise, PERP (p53 apoptosis effector related to PMP22) is an apoptosis-associated target gene of p53 [43], and ATF3 sensitizes human p53-deficient colorectal cancer cells to TNFα-mediated apoptosis [44]. This transcription factor is also required for HDACi-induced apoptosis across different tumor types [45]. A further example relates to platelet factor 4. This cytokine may act via the caspase-mediated extrinsic apoptosis pathway [46] or by inhibition of STAT3 via upregulation of SOCS3 [47]. Moreover, decitabine treatment augments the expression of death receptor 6 (TNF Receptor Superfamily Member 21), and this protein interacts with Bax to induce apoptosis [48]. Furthermore, PBA treatment uniquely induced expression of FAM162A and DUSP6, and FAM162A mediates mitochondrial apoptosis in prostatic and lung adenocarcinoma cells [49, 50], while DUSP6 is a transcriptional target of p53 and regulates p53-mediated apoptosis by dephosphorylating Bcl-2 proteins [51].

Among the commonly upregulated apoptosis genes in response to PBA as well as the combined drug treatment, BCL2 interacting protein 3 (BNIP3) is an interesting example. Indeed, independent research demonstrated the importance of BNIP3 in autophagic cell death [52]. Likewise, the BCL2 interacting protein 3 like mediates p53-dependent apoptosis [53]. Of considerable importance is also the sixfold induced expression of tumor necrosis factor-related apoptosis-inducing ligand (TNFSF10/TRAIL), and it is well known that TNFSF10 induces autophagy [54]. Furthermore, we observed semaphorin 6A upregulated, and recent research profiled SEMA6A as a suppressor of cancer cell migration via the NRF2/HMOX1 axis [55]. Additionally, the bladder cancer-associated protein apoptosis-inducing factor was upregulated, and its overexpression induces S-phase arrest and apoptosis independent of p53 and NFkB [56].

We found the N-Myc downregulated gene 1 > threefold induced in expression, and NDRG1 is required for p53-dependent apoptosis [57]. Conversely, the twofold induced expression of Toll-like receptor 2 signaling mediates the Fas/Fasl-stimulated apoptosis in mouse intestinal epithelial cancer cells [58].

Next to an upregulation of pro-apoptotic genes, we also discovered repression of anti-apoptotic genes, and Fig. 3A3 highlights drug-specific regulations on individual gene expression changes. Decitabine treatment of Caco-2 cells caused repression of the G0/G1 Switch 2, Januskianse 1, angiopoetin 1 and glutamate-ammonia ligase. Note the anti-apoptotic G0/G1 Switch 2 induces cell survival and metastasis through integrin-mediated signaling and interacts with BCL2 [59, 60]. Likewise, inhibition of JAK1 signaling induces apoptosis and cell cycle arrest and reduces tumor cell invasion in colorectal cancer cells [61]. Conversely, angiopoietin-1 is an apoptosis survival factor [62], and its deficiency affects the growth of colorectal cancer liver metastasis [63]. Moreover, DAC treatment of Caco-2 cells repressed the glutamate-ammonia ligase, and this enzyme promotes cell proliferation [64], while glutamine deprivation induces apoptosis [65]. Indeed, stimulating glutaminolysis is an important strategy in the combat of cancer [66].

PBA treatment of Caco-2 cells repressed the expression of DEAD-Box Helicase 3 X-Linked, and DDX3 inhibits apoptosis by forming death antagonizing signaling complex with GSK3 [67]. Unique to the combined decitabine/PBA treatment is the repression of ATPase Family AAA Domain Containing 5, which positively regulates DNA replication, and its depletion increases cell death and genomic instability [68]. Furthermore, the combined decitabine and PBA treatment repressed the ER stress response genes ERP29 and PDIA3. Importantly, ERP29 is frequently deregulated in cancer and forms a feedback loop with microRNA-135a-5p to promote progression of colorectal cancer [69], while protein disulfide isomerase family A member 3 (alias ERP57) modulates folding of glycoproteins and stimulates cell proliferation via c-Myc, PLK1 and the AKT pathway [70]. Moreover, PDIA3 promotes cancer cell proliferation and is associated with poor prognosis in hepatocellular carcinoma [71], while the helicase HELLS functions in DNA-strand separation and replication repair. HELLS is critical for retinoblastoma tumor initiation and progression [72] and is a target of p53 [73]. A further example relates to the combined drug treatment-induced repression of death-associated protein 3, i.e., an essential regulator of the extrinsic pathway for apoptosis resistance and anoikis [74].

### Decitabine and PBA block cell cycle progression

We performed FACS analysis to determine the effects of decitabine and PBA on the cell cycle, and Fig. 3C1 shows the results for three different cancer cell lines. With Caco-2 cells, decitabine treatment resulted in a small but significant increase of cells remaining in the G0/G1 phase and significantly impaired cells progressing from the S-phase (Fig. 3C2). Conversely, PBA alone or the combined PBA/decitabine treatment blocked cell cycle progression, and the cells remaining in the S-phase increased from 36.1 to 89.5% following the combined drug treatment (Fig. 3C2, Additional file 2: Fig. 1SA).

The Caco-2 and the HCT-116 cell lines differed in their response to epigenetic drug treatment. Specifically, with the HCT-116 p53 wild type cell line, DAC treatment caused a significant increase in cells remaining in the G2 phase (from 10.7 to 28.8%), whereas PBA caused an increase in cells remaining in G0/G1 (from 53.7 to 61.6%). We obtained similar results for the combined treatment, i.e., 64.1% are in the G0/G1 phase (Fig. 1H2). Conversely, with the HCT-116-p53-null cell line decitabine treatment caused a significant increase of cells remaining in the S-phase (38.9–53.3%). Moreover, PBA treatment repressed cell cycle progression from the G0/G1 phase (42.2–54.9%) and the combined decitabine/PBA treatment blocked cells progressing from the S-phase (38.9–48.1%) and G2-phase (12.2–46.1%).

Apart from the FACS analysis, we investigated cell cycle regulated genes, and as shown in Fig. 3B1, observed primarily repression of cell cycle regulators following decitabine, PBA and the combined treatment. Depicted in Fig. 3B1 are genes in relation to the different phases of the cell cycle, and PBA treatment caused downregulation of nuclear receptor coactivator 3 (NCOA3) and nuclear factor of activated T cells 5. Note that a recent study highlights the importance of NCOA3 pathways in DNA damage response, and its repression leads to reduced expression of cyclin B1, which results in a G2/M arrest and mitotic catastrophe [75]. In fact, NCOA3 is considered to be a new player in melanoma susceptibility, and in a recent editorial, its function as an activator of cyclins and in-activator of p53, p21 and the cell cycle checkpoint regulator CHK2 was emphasized [76]. Furthermore, the NFAT transcription factors are well appreciated for their functions in the cell cycle and apoptosis [77]. Specifically, NFAT5 promotes cancer progression via transcription of PGK1 [78]. Drug treatment also repressed the expression of G2/M phase-related genes, and next to the obvious ones, i.e., CDK6 and BAX, repression of FUS RNA binding protein and APOC1 following decitabine treatment is a notable finding. Loss of FUS function leads to impaired cellular proliferation and marked increases in phosphorylated histone H3 [79], whereas APOC1 promotes tumor progression via MAPK signaling as shown for colorectal cancer [80] and maintains cell survival by preventing apoptosis [81]. The combined drug treatment also repressed cell cycle regulators, especially the kinesin family member 23 (KIF23) and the protein tyrosine phosphatase non-receptor type 11. KIF23 is a p53 target gene and moves chromosomes during cell division/cytokinesis [82], whereas PTPN11 functions in mitotic cell cycle and oncogenic transformation [83].

### Drug treatment-dependent regulation of tumor-suppressor miRNAs

**Fig. 4:**
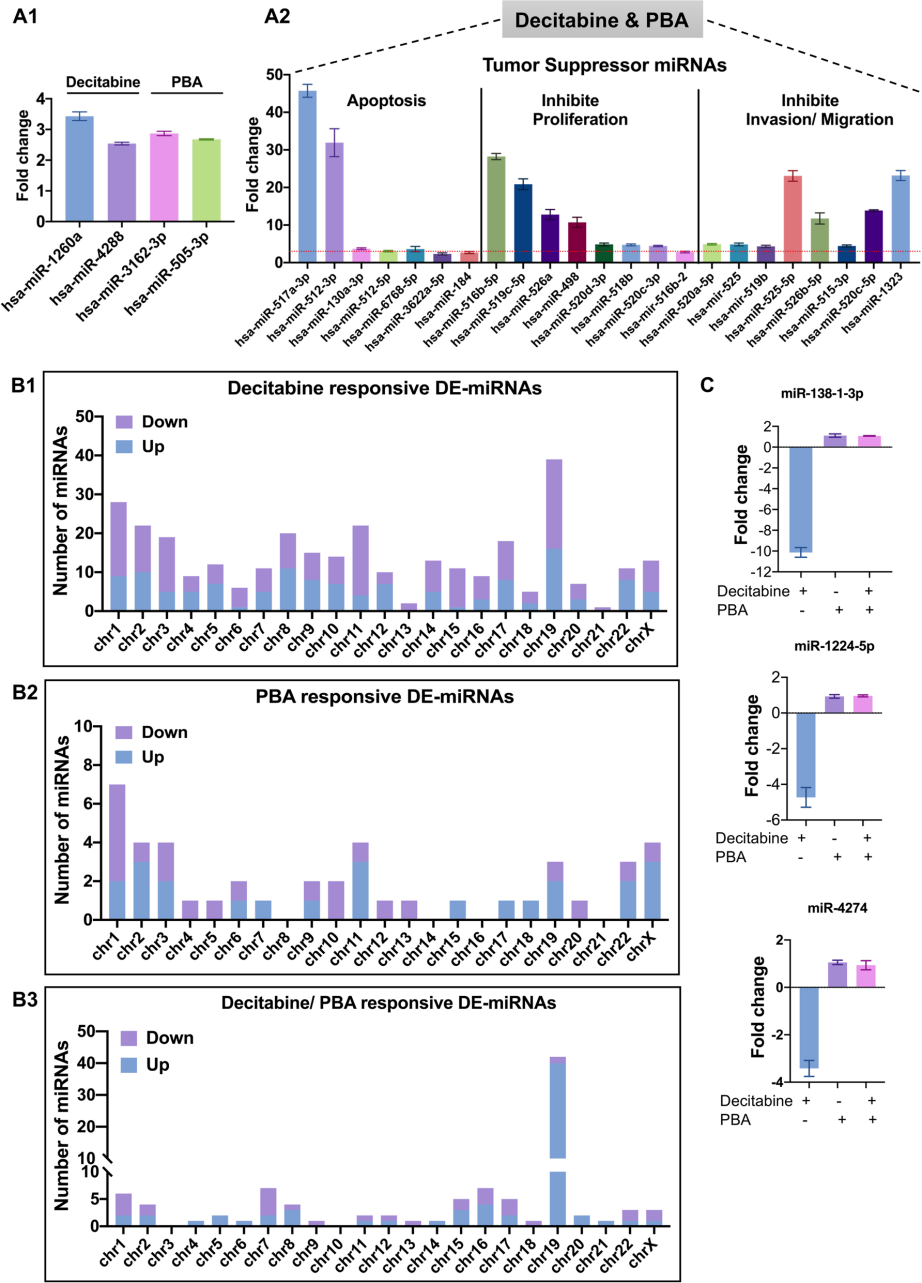
Chromosomal distribution of drug-responsive tumor-suppressor miRNAs. **A** Shown is the upregulation of tumor-suppressor miRNAs induced by the decitabine and PBA treatment of Caco-2 cells (**A1**) and the combined decitabine/PBA treatment (**A2**). Note, 19 out of 23 upregulated miRNAs are located on chromosome 19. **B** The chromosomal distribution of significantly regulated miRNAs following decitabine (**B1**), PBA (**B2**) and the combined drug treatment (**B3**). **C** Repression of tumor-suppressor miRNAs (miR-138–1-3p, -1224-5p and 4274) following decitabine treatment. PBA and the combined decitabine/PBA treatment recovered expression of repressed tumor suppressor miRNAs

Apart from the genomic data, we considered drug treatment-related changes in the expression of ncRNAs. Decitabine, PBA and the combined drug treatment had profound effects on the regulation of miRNAs, and we list significantly regulated miRNAs in Additional file 5: Table S7. We observed 317, 45 and 101 significantly regulated miRNAs of which 130, 24 and 70 miRNAs were up- and 187, 21 and 31 miRNA were downregulated following decitabine, PBA or the combined drug treatment (Additional file 3: Table S2). We focused on > twofold regulated genes and identified tumor-suppressor miRNAs following decitabine or PBA treatment, and the combined decitabine/PBA treatment caused induced expression of 23 tumor-suppressor miRNAs of which 19 (~ 82%) are located on chromosome 19 (Fig. 4A, B) (Additional file 5: Table S7). Strikingly, a previous genetic study identified colon cancer-associated genomic regions on chromosome 19 [84], and nearly 60% of all regulated miRNAs are located on chromosome 19 (Fig. 4B). The tumor-suppressor miRNAs inhibit cell proliferation, invasion and migration. For instance, the combined decitabine/PBA treatment caused 47- and 35-fold induced expression of miRNA 517a-3p and miRNA512-3p (Fig. 4A), and both tumor suppressors promote apoptosis [85, 86]. Likewise, the combined decitabine/PBA treatment of Caco-2 cells caused 29-, 22-, 14- and 12-fold induced expression of miRNA516b-5p, miRNA519c-5p, miRNA526a and miRNA498, and these tumor suppressors inhibit cell proliferation [87–90]. Furthermore, the combined drug treatment caused 22-fold induced expression of miRNA522-5p, and this miRNA reverses drug resistance of doxorubicin-induced HT29 colon cancer cell by targeting ABCB5 [91].

Notwithstanding, decitabine treatment of colon cancer cells also repressed the expression of some tumor-suppressor miRNAs, i.e., 138-1-3p, 1224-5p and 4274. However, PBA alone did not influence their regulation, and the combined decitabine/PBA treatment recovered their expression. The aforementioned miRNAs function in EMT transition and radio-sensitivity [92] as well as cell proliferation and invasion [93, 94]. Moreover, the combined decitabine/PBA treatment caused significant induction of the oncomirs 518f-5p and 518c-5p (Additional file 5: Table S7), and these are located on chromosome 19 and function in proliferation and migration [95, 96]. Given that the combined drug treatment was most effective in inhibition of cell proliferation, i.e., 95%, 90 and 80%, respectively, in Caco-2 cells, HCT-116-p53wt and HCT-116-p53null cells, we consider their regulation as of little relevance.

### Chromosomal map of decitabine and PBA responsive genes

**Fig. 5:**
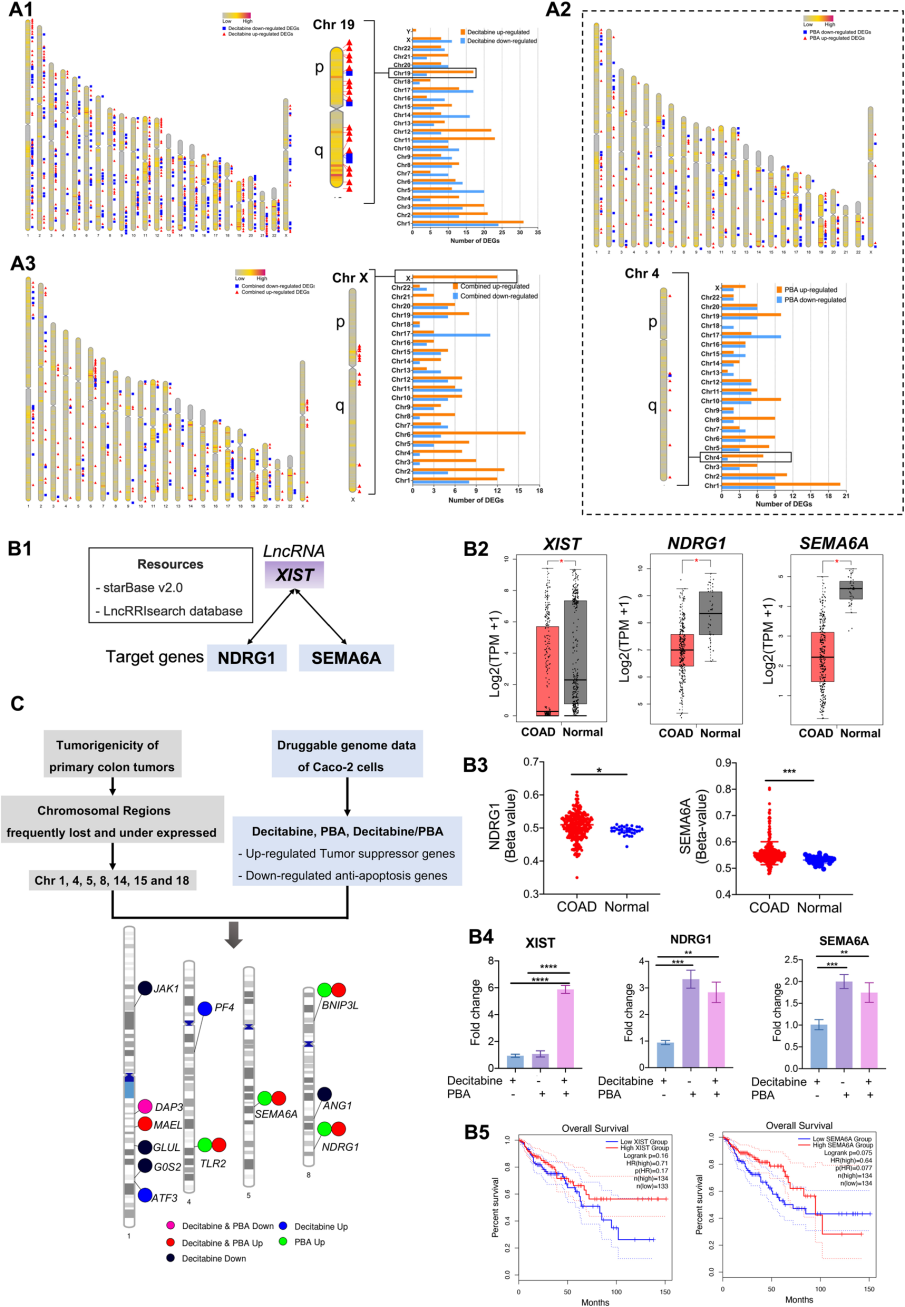
Human chromosomal maps of decitabine and PBA responsive genes. **A** Chromosomal locations of up- and downregulated genes following decitabine (**A1**), PBA (**A2**) and the combined drug treatment (**A3**). The histograms provide a quantitative account of the number of responsive genes on each chromosome. **B** Regulation of the lncRNA XIST and its target genes. Based on the lncRNA–mRNA interaction resource starBase v2.0 and LncRRIsearch, we identified *NDRG1* and *SEMA6A* as target of *XIST* (**B1**). **B2** The gene expression of *XIST, NDRG1* and *SEMA6A* is significantly repressed in COAD tumor samples when compared to histologically proven adjacent normal tissue. The data refer to 275 patients retrieved from the TCGA public repository. **B3** *NDRG1* and *SEMA6A* are hyper-methylated in COAD tumor samples when compared to histologically proven adjacent normal tissue (**B3**). **B4** Regulation of *XIST*, *NDRG1* and *SEMA6A* in Caco-2 cells following drug treatment. *XIST* gene expression is highly dependent on the combined decitabine/PBA treatment, whereas *NDRG1* and *SEMA6A* responded to single PBA treatment. **B5** Kaplan–Meier survival plots for *XIST* and *SEMA6* in low- and high-expression COAD patients. We retrieved the gene expression data of 275 COAD patients from the TCGA public repository, and although not statistically significant, the data imply better survival in high-expression individuals. **C** Drug-responsive tumor-suppressor genes and anti-apoptosis genes are linked to chromosomal hotspots of COAD patients

To define genome-wide responses, we mapped drug-responsive genes along the chromosomes, and Fig. 5 informs on their chromosomal location. We label up- and downregulated genes following drug treatment (Fig. 5A1–A3), and the histograms provide a quantitative account of drug-responsive genes among individual chromosomes. Furthermore, Additional file 6: Table S4 compiles the relative distribution of up- and downregulated genes upon drug treatment. With decitabine, we observed preferentially upregulation of genes localized on chromosome 4, 11, 12, 18, 19 and 21 (ratio up-/downregulated genes > twofold) and is most evident for chromosome 19 (ratio > fourfold). However, there was no clear preference for genes localized on the p- or q-arms of the chromosomes. We performed the same analysis for PBA treatments and identified genes localized on chromosomes 1, 3, 4, 5, 6, 8 and 10 as preferentially upregulated (ratio up-/downregulated genes > twofold). Chromosome 4 can be regarded as a hotspot, and the ratio of upregulated genes is > sevenfold. Although the mode of action of two epigenetic drugs differ, the results for the combined decitabine/PBA treatment is similar to that of PBA, i.e., preferential upregulation of genes on chromosomes 2–6, 8, 14 and especially for genes localized on the X chromosome (ratio 12-fold).

Given decitabine’s mode of action, we investigated the relationship between the number of CpG islands and gene expression changes. Additional file 7: Table S5 compiles all drug-responsive genes and provides information for gene-specific locations of CpG islands, their abundance in promoters and the gene-specific GC content. Based on this information, we computed the sum of CpG islands of upregulated genes and normalized the data by considering the total number of genes for a given chromosome. Subsequently, we determined the average number of CpG islands for upregulated genes and used this value to compare responsive genes among different chromosomes. Essentially, higher CpG values correlated with the number of drug-responsive genes, and this provided a molecular rationale for the differences seen across the chromosomes. It is of considerable importance that the combined decitabine/PBA treatment induced exclusively upregulation of genes localized on chromosome X, and there is growing knowledge on the role of abnormal X chromosome inactivation in tumorigenesis [97–100].

In an earlier study, Tsafrir and colleagues reported the gene expression changes and chromosomal abnormalities in colorectal cancer in a large cohort of patient samples [101]. The study identified highly significant disease-associated transcriptional changes in certain chromosomal regions and established chromosomal hotspots. We were particularly interested whether decitabine and PBA or the combined treatment elicited transcriptional responses in such hotspots, and the results are given in Fig. 5C. Essentially, drug treatment recovered the expression of genes frequently downregulated in primary colon cancer patients. Additionally, we considered the methylation status of individual genes in colon cancer patients using the shiny methylation analysis resource tool (SMART) and show the results for decitabine- and/or PBA-responsive genes across different chromosomes in Additional file 8: Fig. S2. Once again, drug treatment recovered the expression of genes highly methylated in colon cancer.

### Clinical relevance of drug-responsive genes

**Fig. 6:**
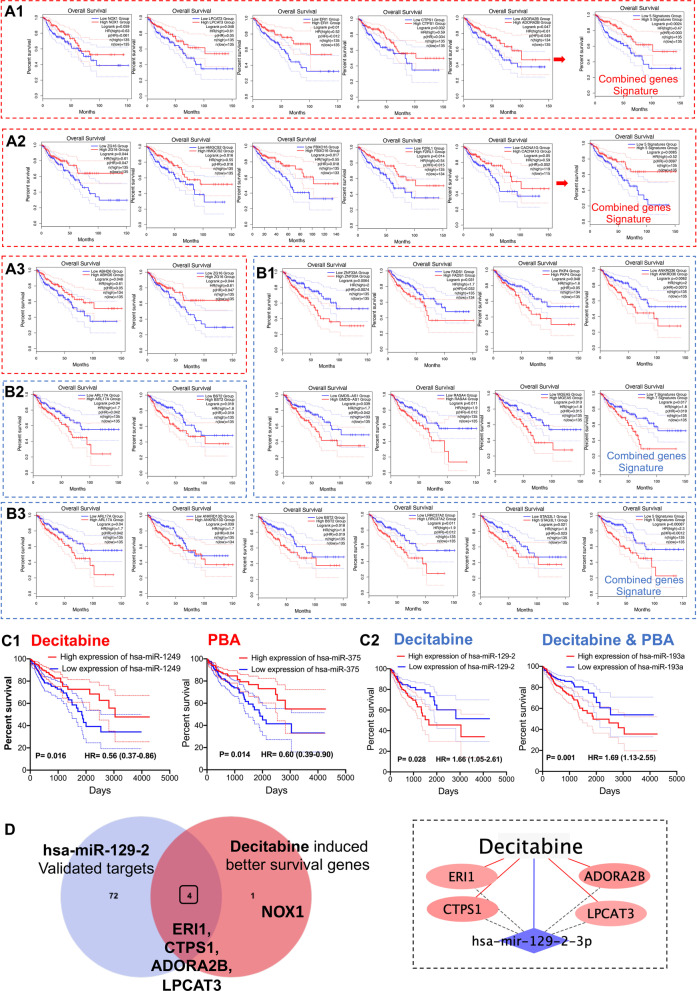
Kaplan–Meier survival plots of drug-responsive genes for 270 COAD patients. Genes either up- or downregulated in colon cancer cells following drug treatment were assessed for their prognostic value in COAD patients. **A1** Shown are Kaplan–Meier survival curves for individual DEGs and the combined genes signature. High expression of decitabine-regulated genes is associated with better survival. The HR ranges between 0.5 and 0.6 and is statistically significant. **A2** Shown are Kaplan–Meier survival curves for individual DEGs and the combined genes signature. High expression of PBA-regulated genes is associated with better survival. The HR ranges between 0.5 and 0.6 and is statistically significant. **A3** Shown are Kaplan–Meier survival curves for individual DEGs. High expression of decitabine-/PBA-regulated genes is associated with better survival. The HR is 0.6 and statistically significant. **B1** Shown are Kaplan–Meier survival curves for individual DEGs repressed by decitabine treatment and the combined gene signature. Lower expression of decitabine-repressed genes is associated with better outcome. The HR ranges between 1.5 and 2 and is statistically significant. **B2** Shown are Kaplan–Meier survival curves for 2 genes repressed by PBA treatment. Lower expression of PBA-repressed genes is associated with better survival. The HR ranges between 1.7 and 1.8 and is statistically significant. **B3** Shown are Kaplan–Meier survival curves for 5 individual DEGs repressed by the combined decitabine/PBA treatment and the combined gene signature. Lower expression of the genes is associated with better survival. For individual genes, the HR ranges between 1.7 and 1.9 and for the combined gene signature is 2.3. **C1** Shown are Kaplan–Meier survival curves for hsa-miR-1249 and hsa-miR-375. High expression of these tumor-suppressor miRNAs is associated with better survival. The HR ranges between 0.56 and 0.6 and is statistically significant. **C2** Shown are Kaplan–Meier survival curves for has-miR-129-2 and has-miR-193a. Lower expression of these oncomirs is associated with better survival. The HR ranges between 1.66 and 1.7 and is statistically significant. **D** Venn diagram of hsa-miR-129-2 validated and decitabine-regulated genes. We queried the miRNet database to search for validated hsa-miR-129-2 target genes and compared the gene targets with decitabine regulated genes. Subsequently, we considered their prognostic value among 270 COAD patients (panel **A1**). The Venn diagram shows the number of unique and common regulated gene targets of hsa-miR-129-2 and decitabine following drug treatment of colon cancer cells for 96 h. High expression of the four common regulated genes is associated with better survival in COAD patients. Decitabine treatment repressed the expression of hsa-miR-129-2, and this resulted in induced expression of four hsa-miR-129-2 target genes. Decitabine treatment caused upregulation of NOX1 independent of hsa-miR-129-2

Based on Kaplan–Meier survival statistics, we assessed the prognostic values of drug-responsive genes for 270 patients (Fig. 6 and Additional file 9: Table S6). We identified 26 drug-responsive genes of clinical relevance whose altered expression in colon cancer cells was associated with better survival of colon adenocarcinoma patients (COAD). The Kaplan–Meier plots shown in Fig. 6A1-6A3 refer to genes induced by decitabine, PBA and the combined drug treatment of Caco-2 cells, and higher expression of the genes was associated with better survival, i.e., the HR ranged between 0.5–0.6. The same consideration applies to Fig. 6B1–6B3. Here we considered genes repressed by the various drug treatments. Although the Kaplan–Meier plots revealed increased HR ratios (range 1.6–2.0), drug treatment repressed their expression, and therefore, they are associated with better outcome. Moreover, we performed Kaplan–Meier plots by combining all drug-responsive genes following decitabine (Fig. 6A1 for upregulated and Fig. 6B1 for downregulated genes) or PBA (Fig. 6A2 for upregulated genes) or the combined drug treatment (Fig. 6B3 for downregulated genes). Occasionally, the gene signatures yielded stronger associations (Fig. 6B3).

Decitabine treatment influenced the expression of five unique genes whose induced expression was associated with better survival (Fig. 6A1). The results for NOX1 were borderline significant, and the protein catalyzes the production of superoxide anions. However, the findings for lysophospholipid acyltransferase 3, exoribonuclease 1, cytidine triphosphate synthase and the adenosine A2b receptor were statistically significant, and the coded proteins function in ferroptosis and programmed cell death, RNA degradation, pyrimidine nucleotide biosynthesis and GPCR signaling.

Likewise, PBA treatment of Caco-2 cells resulted in the regulation of five unique genes whose induced expression was associated with better survival (Fig. 6A2), and this included the zymogen granule membrane protein 16. Typically, ZG16 repressed the expression in colorectal cancer patients though its function is largely unknown [102].

Notwithstanding, a recent study implies a role in host defense immunity and secretory cargo packing of glycoproteins [103]. Additionally, higher expression of 3-hydroxy-3-methylglutaryl-coenzyme A synthase 2 and F-box protein 16 is associated with better survival, and the coded proteins support energy demands and the phosphorylation-dependent ubiquitination, whereas the F2R-like trypsin receptor 1 codes for a protease-activated receptor plays a critical role in inflammation. A further example relates to calcium voltage-gated channel subunit alpha1 G, and its canonical function is the regulation of cell cycle and cell death. Furthermore, the combined PBA/decitabine treatment of Caco-2 cells caused induced expression of the acylglycerol lipase ABHD6 and ZG16, and their higher expression in clinical samples is associated with better survival (Fig. 6A3).

Given its role in the cellular uptake of decitabine and its regulation by PBA, we also investigated the prognostic value of SLC15A1/PEPT1 expression among 440 colon adenocarcinoma patients. We observed high expression of SLC15A1/PEPT1 to be associated with better survival, and the survival plots were nearly significant (p = 0.094, Additional file 2: Fig. S1D).

We also considered the prognostic value of genes repressed by drug treatment of the Caco-2 cell line, and the results are shown in Fig. 6B. For decitabine, PBA and the combined treatment, we identified 7, 2 and 5 genes, respectively, whose reduced expression was associated with better survival in cancer patients. Specifically, zinc finger protein 33 promotes induction of cMyc [104], whereas fatty acid desaturase 1 stimulates AKT/mTOR signaling [105]. Further examples include plakophilin 4, i.e., a protein of desmosomes and the cell–cell adhesion complex known to interact with E-cadherin to support cell migration and to stimulate cMyc activity [106]. Additionally, Ankyrin Repeat Domain 1 supports epithelial–mesenchymal transition and blocks programmed cell death, and its reduced expression in clinical samples was associated with better survival. The physiological role of GMDS divergent transcript is unclear even though its repressed expression is associated with better survival. Conversely, the RAS p21 protein activator 4 is frequently deregulated in malignancies, and the protein functions in the MAPK signaling pathway [107]. Furthermore, lower expression of MGEA5 in clinical samples is associated with better survival and the enzyme removes O-linked N-acetylglucosamine modification of proteins. Its induced expression is associated with larger tumor size, nodal metastases, higher tumor grade and incidence of disease recurrence in human laryngeal cancer [108].

PBA treatment of Caco-2 cells repressed transcript expression of ADP ribosylation factor like GTPase 17A, and this protein takes on diverse roles in mitochondrial biology, whereas the specific functions of bone marrow stromal cell antigen 2 remain uncertain. Nonetheless, their lower expression in clinical samples is associated with better survival.

Finally, the combined drug treatment of Caco-2 cells with decitabine and PBA caused repressed expression of Leucine-Rich Repeat Containing 37 Member A2 and Stromal Antigen 3-Like 1, and the coded proteins function in innate immunity and chromatin binding.

Apart from investigating drug-responsive genes, we considered miRNA-responsive genes following drug treatment. We compared the miRNA genomic profile of Caco-2 cells to 461 COAD tumor samples deposited in the TCGA repository, and this defined 4 miRNAs regulated in common. Shown in Fig. 6C1–2 are Kaplan–Meier plots for miR1249 and miRNA375 whose induced expression is associated with better survival in COAD patients (HR 0.56 and 0.60). Likewise, decitabine and the combined treatment repressed the expression of miR-129-2 and 193a in the Caco-2 cell line, and their reduced expression is associated with better survival. Subsequently, we searched for target genes by querying the miRNet database and identified 4 target genes of clinical significance (Fig. 6D). Thus, decitabine treatment repressed miRNA-129–2-3p, and this resulted in twofold induced expression of ERI1, CTPS1, ADORA2B and LPCAT3 (Additional file 3: Table S2). Importantly, their higher expression is associated with better survival (Fig. 6A1).

### Regulation of DNMT, p53 and histone acetylation by decitabine and PBA

To probe into decitabine and PBA’s mode of action, we performed WB and gene silencing experiments in Cacco-2, HCT-116-p53wt and HCT-116-p53null cancer cells.

**Fig. 7:**
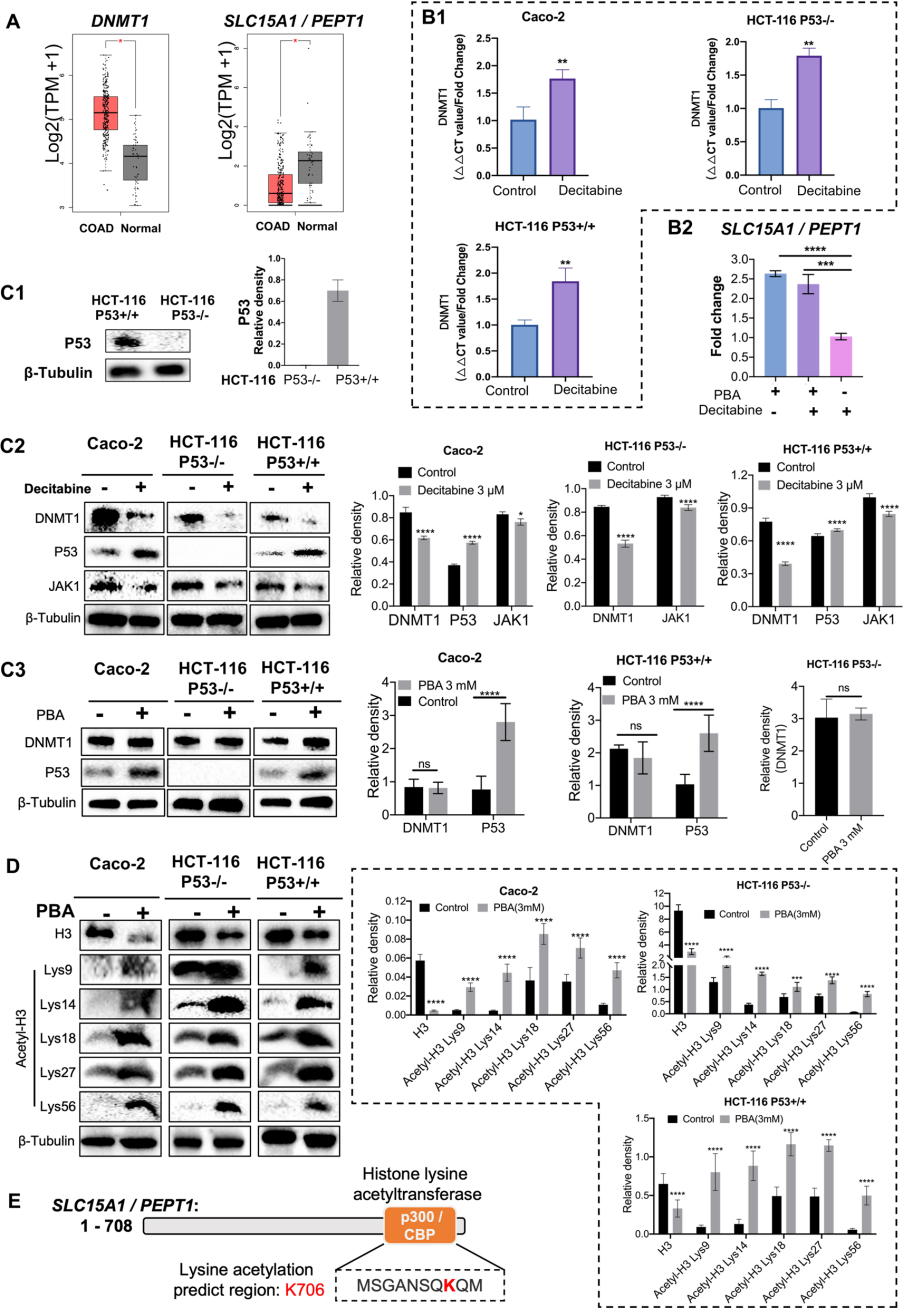
Western immunoblotting of DNMT1, p53, JAK1 and acetylated lysine residues of H3 following decitabine and PBA treatment of colon cancer cell lines. **A** We queried the TCGA public repository and retrieved gene expression data of DNMT1 and the decitabine uptake transporter SLC15A1. We obtained data for 275 COAD patient samples and compared their expression to histologically proven adjacent normal tissue. This revealed DNMT1 to be significantly upregulated in tumor samples; however, SLC15A1 decreased the expression in tumor samples. **B1** Gene expression of DNMT1 in colon cancer cells following decitabine treatment for 72 h. Shown are the results of RT-qPCR assays. Decitabine treatment caused a small but significant increase in DNMT1 gene expression. **B2** Gene expression of the decitabine uptake transporter SLC15A1/ PEPT1 following decitabine, PBA and the combined decitabine/PBA treatment. Treatment of Caco-2 cells with PBA and/or the combined decitabine/PBA treatment caused induced expression of SLC15A. **C1** Western blotting of p53 protein in HCT-116 wild type and p53 null cells. **C2** Western blotting of DNMT1, p53 and JAK1 protein following decitabine treatment of colon cancer cells. Decitabine treatment of colon cancer cells inhibited DNMT1 protein and to a lesser extent the JAK1 protein. Conversely, decitabine treatment of colon cancer cells induced p53 protein expression. The quantification of the WB experiments is shown as histogram. Statistical significance *p < 0.05, **p < 0.01, ***p < 0.001, ****p < 0.0001. **C3** Western blotting of DNMT1 and p53 protein following PBA treatment of colon cancer cells. PBA treatment did not influence DNMT1 protein expression; however, induced p53 protein expression in Caco-2 and HCT-116-p53wt cells. The quantification of the WB experiments is shown as histogram. Statistical significance: ****p < 0.0001. **D** Western blotting of unacetylated and acetylated H3 protein following PBA treatment of colon cancer cells. PBA treatment of Caco-2, HCT-116-p53wt and HCT-116-p53null cells repressed unacetylated H3; however, it strongly induced protein expression of acetylated H3. Shown are immunoblots with antibodies specific for lysine residues 9, 14, 18, 27 and 56 of the H3 protein. The quantification of the WB experiments is shown as histogram. Statistical significance ****p < 0.0001. **E** Acetylation of the decitabine uptake transporter SLC15A1 by CBP/p300. We used the GPS-Pail lysine acetylation prediction tool to identify acetylation sites of the SLC15A1/PEPT1 protein sequence. This defined K706 as a candidate. Independent research confirmed the HDAC inhibitors SAHA and TSA to restore acyltransferase activity of histone lysine acetyltransferase p300 and CBP and to recover SLC15A1 expression in colorectal cancer cells [40]

First, we considered transcript expression of DNMT1 and the cellular uptake transporter of decitabine, i.e., SLC15A1/PEPT1 in clinical samples of 275 tumor and 41 histologically proven normal resection material. While DNMT1 expression was significantly increased in clinical samples, expression of the SLC15A1/PEPT1 transporter was significantly repressed (p < 0.05), Fig. 7A. Second, we assessed DNMT transcript expression (Fig. 7B1) and showed decitabine treatment to cause a small but statistically significant increase in DNMT transcript expression. The increase in DNMT expression did not differ among the three different cell lines, and although decitabine treatment of Caco-2 cells did not alter expression of its drug transporter, PBA alone or the combined decitabine/PBA treatment resulted in induced expression of SLC15A1/PEPT1 (Fig. 7B2). Therefore, PBA treatment supported the expression of the decitabine cellular uptake, and this provides a rationale for the improved potency of the combined drug treatment in inhibiting cell viability and cell proliferation (Fig. 1). Third, we confirmed the absence of the p53 protein in HCT-116-p53 null cells (Fig. 7C1) and evaluated DNMT, p53 and JAK1 protein expression following decitabine treatment. As shown in Fig. 7C2, DAC treatment at a 3 µM repressed DNMT1 and induced p53 protein expression. Furthermore, this treatment caused a small but significant reduction in JAK1 protein expression and agreed with findings from the genomic study where DAC treatment repressed JAK1 transcript expression (Additional file 3: Table S2). Fourth, we evaluated the expression of DNMT1 following PBA treatment, and as expected, PBA did not influence DNMT1 protein expression (Fig. 7C3). However, PBA treatment caused a marked induction of p53 protein in Caco2- and HCT-116-p53 wild type but not in HCT-116-p53null cells (Fig. 7C3).

Given PBA’s mode of action, we investigated histone posttranslational modifications and probed for H3 acetylated lysine residues 9,14,18,27 and 56. Depicted in Fig. 7D are WB experiments and the quantification of three independent experiments. Specifically, PBA treatment of cancer cell lines at 3 mM drug concentrations caused a marked reduction of unacetylated H3 protein, and with Caco-2 cells, this treatment resulted in almost complete ablation of the H3 protein (Fig. 7D). In stark contrast, PBA treatment induced the expression of acetylated H3 protein, as evidenced by immunoblotting of lysine specific residues.

As shown in Fig. 7B2, PBA treatment caused induced expression of SLC15A1/PEPT1, and we predicted acetylation sites of the protein using the GPS-Pail program [109]. We show the functional acetylation sites of SLC15A1/PEPT1 in Fig. 7E, and a recent study provided mechanistic evidence for SLC15A1/PEPT1 activity to be highly dependent on acetylation by the p300 coactivator protein [40].

**Fig. 8:**
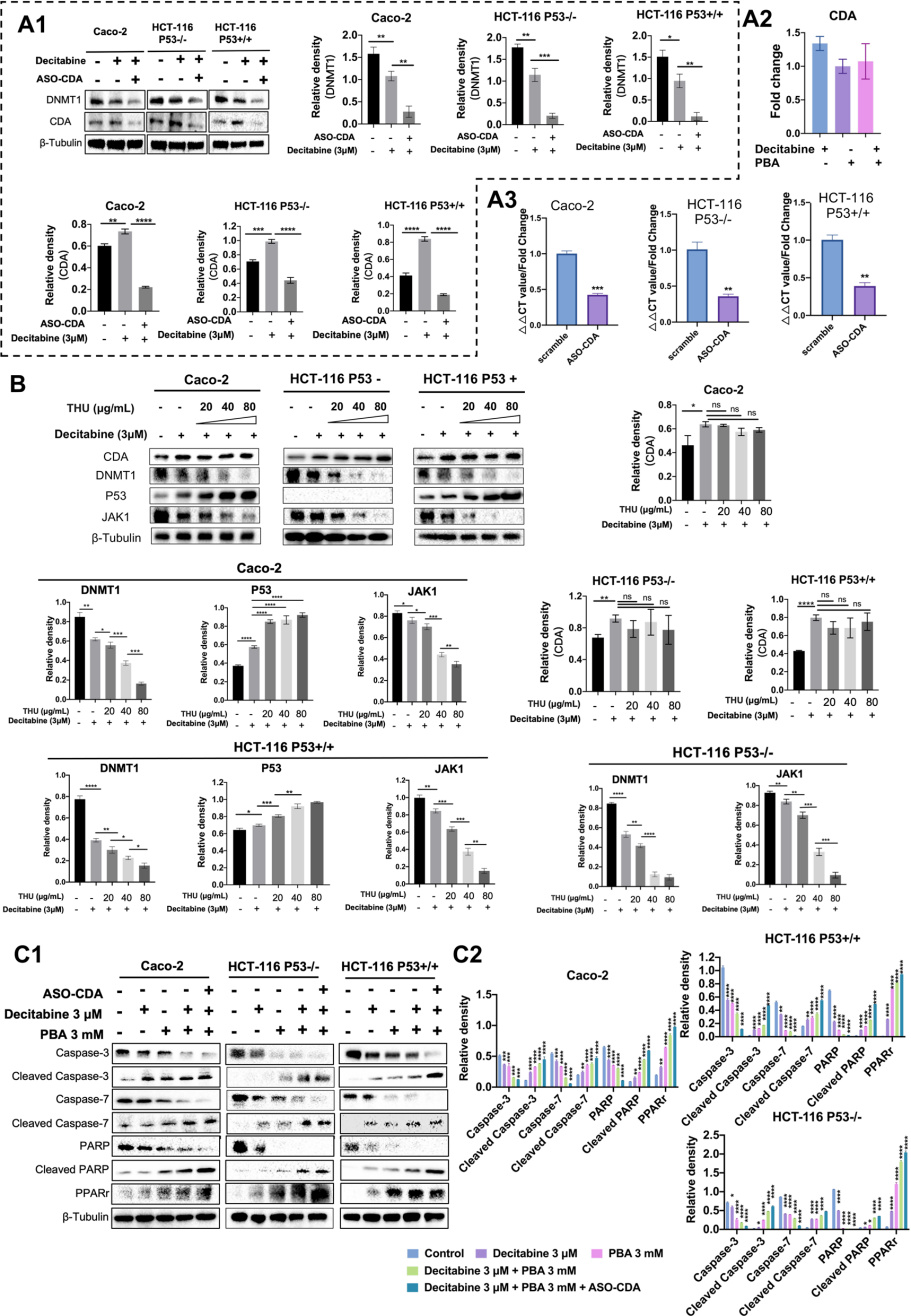

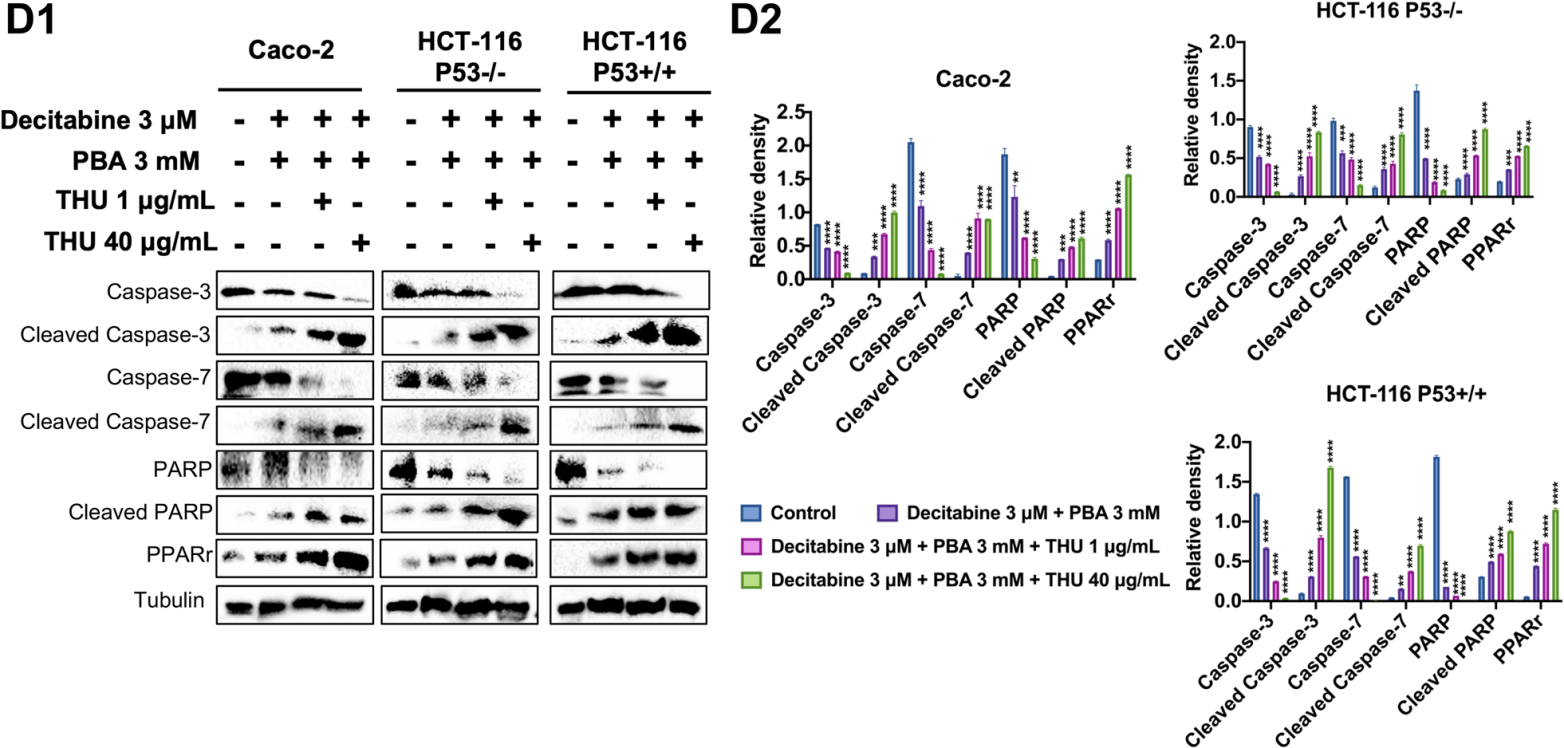
Western immunoblotting of CDA, DNMT1, p53, JAK1, Caspase-3&7, PARP and PPARγ in colon cancer cells following CDA gene knockdown and its pharmacological inhibition by THU. **A1** Western blotting of CDA and DNMT1 following decitabine treatment of colon cancer cells. Decitabine treatment of Caco-2, HCT-116-p53wt and HCT-116-p53null cells repressed DNMT1 protein expression; however, it caused a small but significant induction of the CDA protein. Gene knockdown of CDA in decitabine treated colon cancer cells repressed CDA and DNMT1 protein expression. The quantification of the WB experiments is shown as histogram. Statistical significance: *p < 0.05, **p < 0.01, ***p < 0.001, ****p < 0.0001. **A2** Regulation of CDA gene expression following decitabine, PBA and the combined drug treatment of Caco-2 cells for 96 h. The CDA transcript expression remained unchanged following drug treatment of Caco-2 cells. **A3** CDA gene knockdown in Caco-2, HCT-116-p53wt, and HCT-116-p53null cells. Shown are RT-qPCR assays after treatment of colon cancer cell lines with single-stranded antisense oligonucleotides (ASO) for 72 h. The GapmeRs ASO treatment of colon cancer cells repressed CDA gene expression significantly. Statistical significance: **p < 0.01, ***p < 0.001. **B** Western blotting of CDA, DNMT1, p53 and JAK1 protein after decitabine and THU treatment of Caco-2, HCT-116-p53wt and HCT-116-p53null cells for 72 h. We used 20, 40 and 80 μg/ml THU drug concentrations to inhibit CDA activity in decitabine treated colon cancer cell lines. Decitabine at 3 µM concentrations caused a small but significant increase in CDA protein. Importantly, drug treatment induced p53 and repressed DNMT1 and JAK1 protein expression. The combined decitabine/THU treatment at various drug concentrations had no effect on CDA; nonetheless, significantly induced p53 and repressed JAK1 protein expression. The quantification of the WB experiments is shown as histogram. Statistical significance: *p < 0.05, **p < 0.01, ***p < 0.001, ****p < 0.0001. **C** Immunoblotting of Caspase-3, -7, PARP and PPARγ protein in response to decitabine, PBA and the combined drug treatment of colon cancer cells for 72 h. Decitabine treatment of Caco-2, HCT-116-p53wt and HCT-116-p53null cells repressed Caspase-3, -7 and PARP protein; however, induced expression of the cleaved products and PPARγ protein. Thus, decitabine treatment induced programmed cell death. Likewise, PBA treatment of colon cancer cells significantly repressed Caspase-3, -7 and PARP protein expression; however, it induced the cleaved products and PPARγ protein (**C1**). Importantly, in CDA silenced colon cancer cell lines, the effects of the combined decitabine/PBA treatment on apoptosis were enhanced. The quantification of the WB experiments is shown as histogram (**C2**). Statistical analysis: *p < 0.05, **p < 0.01, ***p < 0.001, ****p < 0.0001. **D** Immunoblotting of Caspase-3&7, PARP and PPARγ following the combined decitabine/PBA and THU treatment of colon cancer cells. The combined decitabine/PBA treatment of Caco-2, HCT-116-p53wt and HCT-116-p53null cell lines repressed expression of caspase-3&7 and PARP protein; however, it induced expression of the cleaved products and PPARγ. Thus, decitabine/PBA treatment induced programmed cell death (**D1**). Pharmacological inhibition of CDA at two different THU drug concentrations significantly enhanced the effects of combined decitabine/PBA treatment. The quantification of the WB experiments is shown as histogram (**D2**). Statistical analysis: *p < 0.05, **p < 0.01, ***p < 0.001, ****p < 0.0001

We already emphasized the fact that decitabine is rapidly deaminated by cytidine deaminase (CDA), which causes an inactivation of the drug. Therefore, we performed gene knockdown studies to repress CDA transcript expression in decitabine-treated colon cancer cells. As shown in Fig. 8A1, gene knockdown of CDA resulted in marked inhibition of DNMT1 protein following decitabine treatment of colon cancer cells, and the results of 3 independent experiments are given as histograms (Fig. 8A1). Although decitabine treatment of colon cancer cells repressed DNMT1 protein expression by about 30% (Fig. 8A1), the additional gene knockdown of CDA enhanced decitabine’s ability to promote DNMT1 protein degradation, and depending on the cell line, its expression was reduced between 80 and 90%.

Notwithstanding, neither decitabine nor PBA or the combined drug treatment influenced CDA transcript expression in Caco-2 cells (Fig. 8A2), and based on RT-qPCR assays we achieved about 60% reduction of CDA mRNA in gene knockdown studies (Fig. 8A3). The results imply that blocking CDA is of critical importance to support DNMT degradation.

Additionally, we employed a pharmacological approach to inhibit CDA and evaluated the effects of the CDA inhibitor tetrahydrouridine (THU) on DNMT1 protein expression. We investigated three different THU drug concentrations of which the 40 µg/ml is clinically relevant, i.e., Cmax concentrations as determined in patients diagnosed with malignant melanoma [110]. We treated the cancer cell lines daily with THU for 48 h, followed by the combined decitabine and THU treatment for another 48 h. We showed the WB experiments of DNMT1, p53 and JAK1 in Fig. 8B and observed a clear dose-related repression of the DNMT and JAK1 protein. Strikingly, the combined decitabine and THU treatment is highly effective in ablating DNMT1 protein expression at 40 and 80 µg/ml THU drug concentrations, even though THU itself had no effect on CDA protein expression. THU inhibits CDA activity, and the combined THU and decitabine treatment of cancer cells was similarly effective in inhibiting JAK1. However, Caco-2 cells appeared less sensitive. Furthermore, the combined decitabine and THU treatment caused strong induction of p53 in the Caco-2 and HCT-116-p53 wild-type cells.

Additionally, we investigated the effects of decitabine and/or PBA on programmed cell death and studied the regulation of caspase 3 & 7, PARP and PPARγ after daily treatment of the cancer cell lines for 72 h and in CDA gene knockdown cell lines. Note the role of PPARγ-induced apoptosis in cancer cells has been the subject of reviews [111], and depicted in Fig. 8C1–C2 are the results of WB experiments. When compared to the combined treatment, decitabine or PBA alone was less effective in inducing cleaved caspase 3, 7, cleaved PARP and PPARγ expression. Notwithstanding, we observed opposite regulation between the parent proteins and their cleaved products, and the best results were obtained in CDA gene knockdown cell lines following the combined decitabine/PBA treatment. Collectively, we obtained strong evidence for the combined drug treatment to induce cell death and confirmed the importance of CDA inhibition for an improved cell death response following decitabine treatment.

We also investigated the effects of THU on programmed cell death following the combined decitabine/PBA treatment (Fig. 8D1, D2). Initially we treated the cancer cell lines at 1 and 40 µg/ml THU for 48 h followed by the combined decitabine (3 µM)/PBA (3 mM) treatment for another 48 h. THU improved the drug treatment effects dose-dependently, and even the lower dose of 1 µg/ml THU enhanced the expression of cell death inducing proteins (Fig. 8D2).

**Fig. 9:**
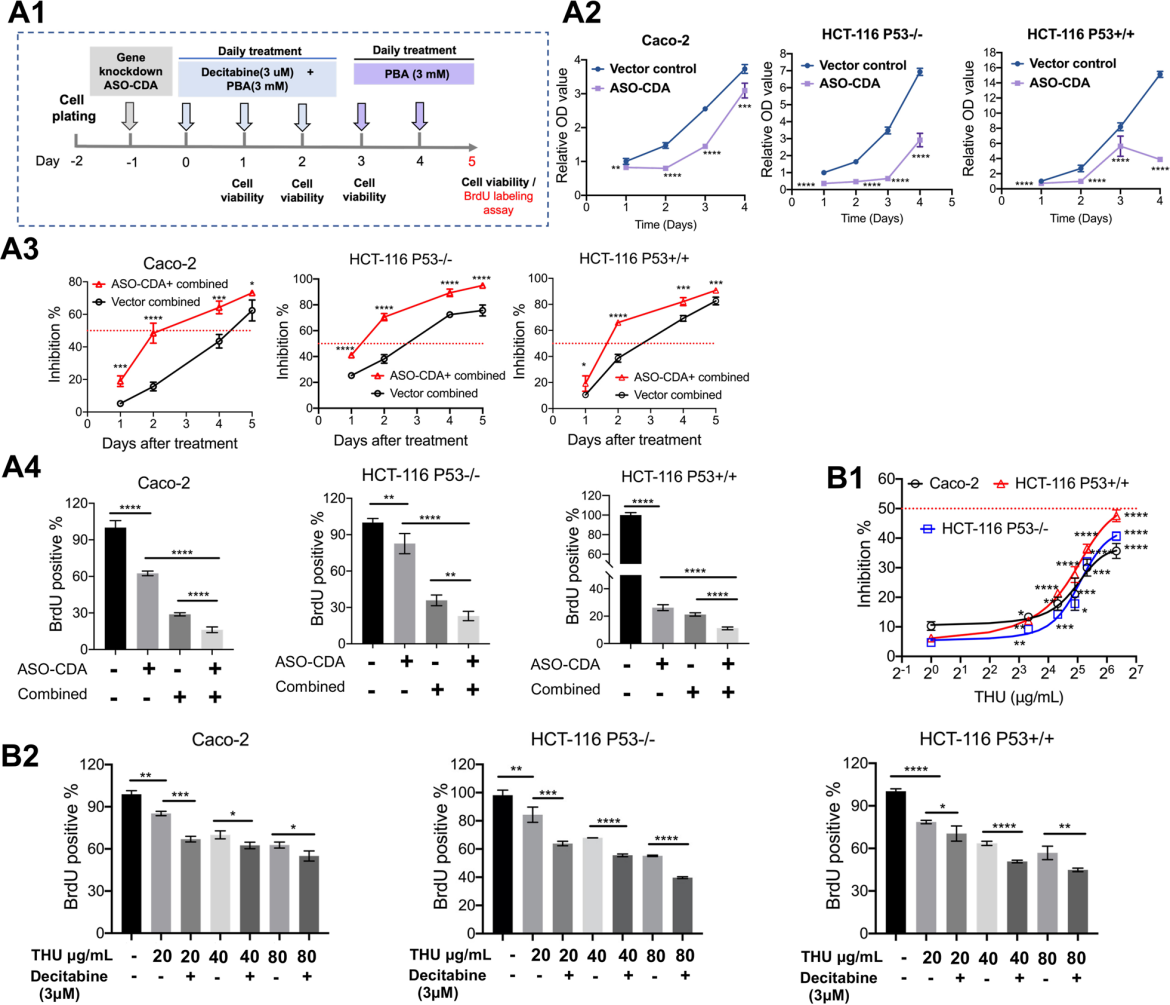
The effects of decitabine/PBA and THU on cell proliferation in CDA silenced colon cancer cell lines. **A1** Treatment scheme. We treated Caco-2, HCT-116-p53wt and HCT-116-p53null cells with the single-stranded antisense oligonucleotides (ASO) for 24 h followed by the combined decitabine/PBA drug treatment for 5 days. **A2** The effects of CDA gene knockdown on cell proliferation of colon cancer cell lines. Shown are the relative OD value of Caco-2, HCT-116-p53wt and HCT-116-p53null cells following single CDA gene knockdown. Gene silencing of CDA inhibited cell viability when compared with vector controls. Statistical analysis: *p < 0.05, **p < 0.01, ***p < 0.001, ****p < 0.0001. **A3** Inhibition of cell proliferation in CDA silenced colon cancer cell lines following combined decitabine/PBA treatment. CDA gene knockdown improved therapeutic efficacy of the combined decitabine/PBA treatment significantly and caused 70–95% inhibition of cell viability among the three colon cancer cell lines. Statistical analysis: *p < 0.05, **p < 0.01, ***p < 0.001, ****p < 0.0001. **A4** DNA synthesis in CDA silenced colon cancer cell lines. CDA gene knockdown of colon cancer cell lines inhibited DNA synthesis as determined by the BrdU labeling assay. Depending on the cell line, the inhibition of DNA synthesis ranged between 20% (HCT-116-p53null) to 70% (HCT-116-p53wt). Additional decitabine/PBA treatment was most effective in inhibiting DNA synthesis (range 80–90%). Statistical analysis: *p < 0.05, **p < 0.01, ***p < 0.001, ****p < 0.0001. **B1** Inhibition of cell proliferation of colon cancer cell lines following daily THU treatment at different drug concentration for 48 h. We treated Caco-2, HCT-116-p53wt and HCT-116-p53null cells with various drug concentrations of the CDA inhibitor THU. At 80 µg/ml THU the inhibition of cell proliferation ranged between 35 and 48%. Statistical analysis: *p < 0.05, **p < 0.01, ***p < 0.001, ****p < 0.0001. **B2** Effects of various THU drug concentrations on DNA synthesis in colon cancer cell lines. We treated Caco-2, HCT-116-p53wt and HCT-116-p53null cells with varying concentrations of the CDA inhibitor THU. At 80 µg/ml THU the inhibition of DNA synthesis ranged between 30 and 40%. The combined THU /decitabine treatment of colon cancer cells was more effective in inhibiting DNA synthesis (range 40–60%). Statistical analysis: *p < 0.05, **p < 0.01, ***p < 0.001, ****p < 0.0001

Furthermore, we investigated cell proliferation of cancer cell lines following CDA gene knockdown. We showed the treatment scheme as well as the results for the MTT assay in Fig. 9A1, A2 and observed a significant reduction in cell proliferation following single treatment of the cancer cell lines with the CDA antisense oligonucleotide for 24 h. Thus, CDA gene knockdown alone inhibits cell proliferation; however, the response varied between the three cell lines and was pronounced with p53 functional HCT-116 cells. Subsequently, we investigated the effects of the combined decitabine & PBA treatment on cell proliferation in CDA gene knockdown cell lines. Strikingly, the combined drug treatment caused marked inhibition of cell proliferation, and in the case of the p53 null cell line reached nearly 100% (Fig. 9A3, A4). We confirmed the results of the MTT assay in BrdU labeling studies and once again achieved almost complete inhibition of cell proliferation following the combined drug treatment.

Finally, we investigated the effects of various THU drug concentrations on cell proliferation (Fig. 9B1. At 80 µg/ml THU, the inhibition of cell proliferation ranged between 35 and 48%. Here the statistical significance relates to a comparison of drug responses among the three different cell lines, and the HCT-116-p53 wild-type cell line displayed higher sensitivity to THU drug treatment effects. Subsequently, we corroborated the results with the BrdU labeling assay, and the combined THU decitabine treatment is more potent in inhibiting cell proliferation (Fig. 9B2). Therefore, CDA inhibition improved DAC’s potency in inhibiting cell proliferation.

## Discussion

Targeting the epigenome represents an innovative approach for the treatment of cancerous diseases [112], and in an effort to develop an epigenetic therapy of colorectal cancer, we investigated the effects of the DNA methylation inhibitor decitabine and the histone deacetylase inhibitor PBA on a genome-wide scale. Epigenetic drug treatment of cancer cell lines caused marked inhibition of cell proliferation, and induction of programmed cell death while genome-wide scans informed on the significant upregulation of tumor-suppressor genes and of genes coding for the pro-apoptotic pathways. Furthermore, the genomic study revealed drug treatment-related repression of anti-apoptotic genes as well as genes coding for cell cycle progression, e.g., cyclin-dependent kinases and genes coding for cytokinesis. Moreover, drug treatment induced an unprecedented induction of 23 miRNAs, which function as tumor suppressors, and we confirmed the genomic results by evidencing cleaved caspases 3 and 7, and PARP, and inhibition of cell proliferation. Additionally, we show drug treatment-related inhibition of cell proliferation by cell cycle analysis and fluorescent microscopy of EdU-labeled colon cancer cells. Together, drug treatment induced programmed cell death, and we identified drug-responsive genes causing impaired cell cycle progression especially in the S- and G2M phase (Fig. 3B1). Strikingly, the combined decitabine/PBA treatment was most effective in an inhibition of cell proliferation and the reduction of cell viability (range 80–95% for the three different cell lines, Fig. 1). Moreover, the genomic study enabled us to decipher the functional significance of regulated genes, and based on gene ontologies we grouped them into distinct pathways (Fig. 2A–C). Prominent examples included regulation of histone acetylases and transcriptional regulation by p53, chromatin organization, chromosome maintenance and segregation, CENP-A nucleosome and kinetochore formation, RNA polymerase I promoter opening, as well as various signaling pathways induced by decitabine, PBA or the combined drug treatment.

We mapped drug-responsive genes to human chromosomes, and for the repressed ones, did not observe chromosomal hotspots nor did we observe a predilection of genes localized either on the p- or q-arm of chromosomes (Fig. 5). However, decitabine treatment of cancer cell lines specifically induced the expression of genes localized in cancer-associated genomic region of chromosomes 19, and the ratio of upregulated genes versus repressed ones is > 4 and accounted for 6% of all upregulated genes (Additional file 6: Table S4). Moreover, PBA treatment preferentially induced genes localized on chromosome 4. Here the ratio of upregulated versus repressed ones is > 7 and accounted for 5.2% of all upregulated DEGs. Finally, the combined decitabine/PBA treatment induced exclusively upregulation of genes on the X chromosome.

We considered the GC content for each chromosome, and noticed the highest GC content for chromosome 19 (48.4%). This provided a molecular rationale for the marked upregulation of genes following decitabine treatment. However, for chromosome 4 and the X-chromosome, the GC content was similar to the other ones (Additional file 6: Table S4). Therefore, other epigenetic mechanisms may be functional. The unique upregulation of genes localized on the X chromosome and especially of the long ncRNA XIST is of considerable importance. Specifically, there is growing evidence for a direct relationship between X-chromosome inactivation (XCi) and tumorigenesis [97], and a very recent report demonstrated the importance of XIST in human mammary epithelium homeostasis [113]. Indeed, failure of XIST expression impairs mammary stem cell differentiation and increases tumorigenicity through hyperactivation of the mediator coactivator multi-protein complex [113]. Following the combined decitabine/PBA treatment of colon cancer cells, we observed a highly significant sixfold induced expression of XIST and a three- and twofold induction, respectively, of its target genes NDRG1 and SEMA6A. These function in p53-mediated apoptosis and as tumor suppressor. Moreover, we demonstrated XIST and its targets NDRG1 and SEMA6A to be significantly repressed in clinical samples (Fig. 5B2) and provided evidence for hypermethylation of NDRG1 and SEMA6A in colon cancer clinical samples (Fig. 5B3).

The unique upregulation of 12 genes localized on the X-chromosome is an important finding, and the X-chromosome inactivation (XCi) requires specific proteins, i.e., SAFA, LBR and SHARP. Their interaction with XIST is essential for XCi (94). Apart from XIST, the combined decitabine/PBA treatment caused induced twofold and threefold expression of the tudor domain containing 9 and 12. These RNA binding proteins are important epigenetic regulators, which function as RNA helicase and endorse gene silencing. Moreover, tudor domain containing proteins recognize histone modifications and function as adaptor proteins of histone. Indeed, the Lamin B receptor (LBR) contains various tudor domain containing proteins of the inner nuclear envelope membrane proteins, and these are known to interact with XIST.

Strikingly, we observed an unprecedented up to 47-fold induced expression of tumor-suppressor miRNAs primarily localized on cancer-associated genomic regions of chromosome 19 (Fig. 4), and this demonstrates the great therapeutic potential of epigenetic drugs in reprogramming cancer genomes. Indeed, a previously performed cancer genomic study of a large cohort of colorectal cancer patients identified disease-associated transcriptional changes in certain chromosomal regions [101]. We were particularly interested to investigate the effects of epigenetic drug treatment and showed many genes localized on colorectal cancer-related chromosomal hotspots to be responsive to decitabine, PBA and the combined drug treatment (Fig. 5 and Additional file 8: Fig. S2). Among the chromosomes 1, 4, 5, 8, 14, 15 and 18, we identified 12 genes coding for tumor-suppressor and anti-apoptosis genes, which were specifically regulated by the epigenetic drug treatment (Fig. 5C), and all of these genes are localized on chromosomal hotspots of COAD patients.

To determine clinical relevance, we computed Kaplan–Meier survival plots, and for 26 drug-responsive DEGs, we established better outcome based on the cancer genomic data of 270 COAD patients (Fig. 6). Therefore, the findings obtained from the colon cancer cell lines are of prognostic relevance for COAD patients, and similar results were obtained for 4 miRNAs regulated by the epigenetic drugs.

Importantly, earlier studies with epigenetic drugs failed to show efficacy in solid tumors [114], yet the combination of epigenetic drug treatment with other therapeutic approaches carry the hope for a successful genome medicine approach. Indeed, “reprogramming” cancer cells at the genome level are a promising therapeutic intervention, and in the present, we show the combined use of decitabine and PBA to be excellent epigenetic modifiers by blocking undue hypermethylation and deacetylation of histones.

In regard to clinical trials, and starting from the year 2003, we identified 16 studies, which evaluated the therapeutic efficacy of the combined use of DNA demethylating agents and histone deacetylase inhibitors in different cancer patient cohorts. Given the primary objective of our study, we focused on solid tumors and identified three studies involving 17, 13 and 45 lung cancer patients (NCT01935947, NCT01207726, NCT00387465). Unfortunately, these studies are not directly comparable as they included patients on various chemotherapeutics given either before or after the epigenetic treatment cycle. Furthermore, the interpretation of the findings is confounded by factors such as differences in the clinical stages of patients and the treatment rationale, i.e., curative versus palliative. Nonetheless, the data are suggestive for a potential improved outcome based on the pathological response, i.e., RECIST v1.1 criteria as well as overall survival. Another study involved patients with metastatic melanoma and the combined treatment of decitabine, the HDAC inhibitor panobinostat and the cytostatic agent temozolomid. In this study, 30% of patients remained stable, but 70% displayed progressive disease during an observation period of 3–4 years (NCT00925132). Lastly, there are two studies on colorectal cancer. Specifically, one study (NCT01105377) involved metastatic colorectal cancer patients classified at clinical stage 4. The study population consisted of two study cohorts of 24 and 23 patients, and the cohorts received the combined treatment of azacytidine and the HDAC inhibitor entinostat. The primary outcome was tumor regression, and based on RECIST criteria the mean time for disease progression ranged 1.8 to 1.9 months among the two study cohorts. The second study is a phase I dose range-finding study of combined 5-azacytidine and PBA. The study enrolled 27 patients with advanced solid tumors (NCT00005639), and the investigators confirmed the inhibitory activity of PBA on histone deactylases by Western immunoblot analysis in peripheral blood monocytes (PBMCs) [115]. However, only seven out of the 27 patients are CRC cases, and the primary objective was to investigate pharmacokinetic and pharmacodynamics parameters following the combined use of decitabine and PBA. While the efficacy data were daunting, the lack of clinical response is linked to the fact that patients received four different treatment cycles of various drug cocktails prior to their study enrollment. Moreover, the patients received on average only 1.7 cycles of the epigenetic drug cocktail.

Moreover, we identified five clinical trials involving the CDA inhibitor THU combined with decitabine in patients with solid tumors; however, none involved CRC patients. Four of the five studies are completed, and trial NCT00359606 recruited 58 patients with advanced solid tumors. Here one refractory breast cancer patient experienced > 90% regression in tumor size and the partial response lasted over 1 year [116]. Another phase II study recruited 95 patients with various solid cancers, and best results were obtained for bladder cancer patients with a progression-free survival (PFS) rate of 42% during a 4-month observation period (NCT00978250).

Additionally, the effects of THU and decitabine were investigated in 13 patients diagnosed with advanced chemo-refractory pancreatic cancer (NCT02847000). The study highlighted major differences in CDA plasma activity when cases of metastatic versus resectable pancreatic ductal adenocarcinoma were compared [117]. In this pilot study, sub-therapeutic THU dosages were used, and no therapeutic benefit could be demonstrated.

Moreover, Savona et al. investigated an oral fixed-dose combination of decitabine and cedazuridine, i.e., a CDA inhibitor, in patients with myelodysplastic syndromes or chronic myelomonocytic leukemia [17] and demonstrated favorable decitabine PK through inhibition of CDA. Furthermore, a mass balance and metabolite profiling study of 14C-guadecitabine in patients with advanced cancer evidenced that most of the administered dose is excreted as degradation products/metabolites [118, 119]. In fact, only 0.3% of the dose was recovered as ß-decitabine, and this shows the importance of CDA inhibition in blocking guadecitabine metabolic inactivation.

In an effort to identify potential mechanisms of epigenetic drug failures, we investigated the regulation of DNMT1 by decitabine and its inactivation by cytidine deaminase. Blocking cytidine deaminase supported degradation of the DNMT1 protein in cancer cell lines (range 82–90%, Fig. 8), even though decitabine treatment alone of cancer cells caused a small but significant increase in DNMT1 mRNA expression (Fig. 7B). Associated herewith, we demonstrated induction of programmed cell death by immunoblotting of cleaved caspase, PARP and induced PPARγ protein (Fig. 8). Finally, blocking CDA activity pharmacologically or by gene knockdown was most effective in inhibiting cell proliferation following the combined decitabine/PBA treatment as evidenced by the MTT (inhibition range 70 – 95%) and the BrdU-assay (inhibition range 70–90%, Fig. 9). However, inhibition of CDA activity by THU did not influence CDA protein expression (Fig. 8). Nonetheless, at supra-therapeutic drug concentrations, THU inhibited cell proliferation of cell lines up to 50% (Fig. 9B).

Another important finding of our study is the highly significant upregulation of the decitabine uptake transporter SLC15A1 following PBA or the combined decitabine/PBA drug treatment (Fig. 7). Thus, PBA supports the intracellular loading of decitabine in colon cancer cells.

**Fig. 10:**
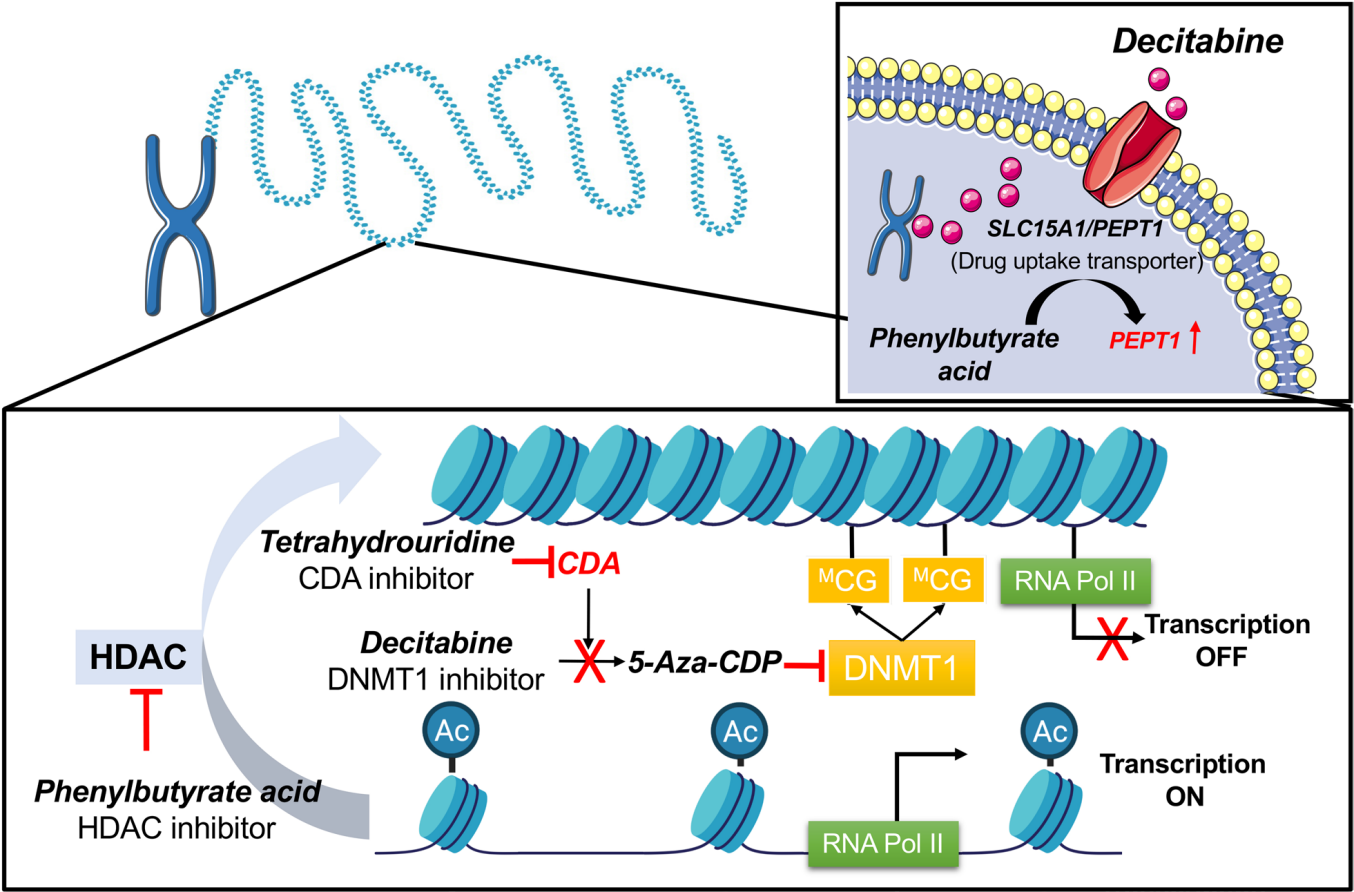
Mechanism of decitabine, PBA and THU drug treatment of cancer cell

Figure 10 summarizes the mechanistic aspects and the pharmacological rationale of a therapeutic scheme consisting of the decitabine, PBA and THU for the treatment of colon cancer. We show PBA to be most effective in reinstalling H3 acetylation, and this supports an open chromatin state and accessibility for RNA polymerase for gene transcription, while decitabine inhibits activity of the DNA methyltransferase. Although cytidine deaminase inactivates decitabine activity on DNMT, blocking CDA’s activity with THU improved decitabine’s efficacy considerably. Strikingly, PBA specifically induced expression of the decitabine uptake transporter SLC15A1, and this improved intracellular loading of tumor cells for sustained tumor cell eradication.

As discussed before, decitabine drug treatment of solid tumors failed in clinical trials, and this can be attributed, at least in part, to its rapid deamination by cytidine deaminase. Here we propose the pharmacological inhibition of CDA by THU to significantly improve therapeutic efficacy of decitabine, and the combined decitabine/PBA treatment caused nearly 100% inhibition of cell viability and up to 90% inhibition of cell proliferation based on the BrdU labeling of cells (Fig. 9A4). Thus, targeting DNMT and histone deacetylases at the same time is highly effective, providing that the deamination of decitabine is prevented by inhibiting CDA activity.

## Conclusions

Our study provides a molecular rationale for first-line treatment of colon cancer patients with a combination of DNMT, CDA and histone deacetylase inhibitors. Given their existing approval for various indications, the clinical testing of the triple combination is warranted to demonstrate therapeutic efficacy.

## Methods

### Cells and cell culture

We obtained the human colon cancer cell line (Caco-2) from American Type Culture Collection (ATCC), and the HCT-116-p53wt and the HCT-116-p53null cell lines were the kind gift of Dr. Vogelstein (Genetics Resources Core Facility Johns Hopkins University Baltimore, USA).

The cell lines were tested negative for mycoplasma contamination and cultured in DMEM (Gibco, USA) supplemented with 10% heat-inactivated fetal bovine serum (Gibco, USA), 100 µg/ml penicillin and 100 U/ml streptomycin (Gibco, USA) in a 5% CO2 atmosphere at 37 °C.

### Epigenetic drug treatment of cell cultures

Decitabine (5-Aza-2′-deoxycytidine) was sourced from Sigma-Aldrich, Germany, and dissolved in DMSO. Based on clinical trials, the therapeutic drug concentration is about 3 µM, and therefore, we chose concentrations below and above Cmax therapeutic drug concentrations [37]. We performed cellular assays at 0.5, 1, 3, 6, 12, 25 µM.

4-Phenylbutyric acid (4-PBA) was purchased from Sigma-Aldrich, Germany. We performed cellular assays at 0.5, 1, 3, 6, 12 mM, and based on clinical trials, a relevant Cmax drug concentration is 1.878 mM [38].

The competitive cytidine deaminase inhibitor THU (Tetrahydrouridine) was purchased from MERCK, Germany, and assays were performed at 1, 10, 20, 30, 40, 80 µg/ml, respectively.

### RT-qPCR of DNMT1 and CDA

We assayed the expression of DNMT1 and CDA in treated cells by reverse transcription quantitative PCR (RT-qPCR) and used GAPDH as a normalizer (housekeeping gene). We used the miRNeasy Mini Kit (QIAGEN, Germany) to isolate total RNA according to the manufacturer’s recommendations and performed cDNA synthesis on a C1000 Touch Thermal Cycler (Bio-Rad, USA) following the manufacturer’s protocol of the Omniscript Reverse Transcriptase Kit (QIAGEN, Germany). Typically, we initiated cDNA synthesis with 500 ng to 1 µg template RNA. We performed qPCR of target genes on a CFX96 Real-Time System (Bio-Rad, USA). The master mix consisted of 10 ng cDNA template (0.1 µl), 100 nmolar primers (1 µl), nuclei acid-free water (3.9 µl) and 5 µl SsoAdvanced™ Universal SYBR® Green Supermix (Bio-Rad, USA). The total qPCR assay volume is 10 µl, and we calculated the expression levels of target genes by the delta-delta CT method. The primers for DNMT1 and CDA were purchased from Eurofins, Germany, and the sequences are given in Additional file 10: Table S8. On average three independent experiments were assayed in duplicate measurements.

### Cell viability and proliferation assays

#### MTT assay

We seeded 2 × 10^3^ cells of Caco-2, HCT-116-p53wt and HCT-116-p53null cells into 96-well plates and performed the assay in a final volume of 100 µL of culture media. We performed the MTT assay to determine cell viability following drug treatment and purchased the tetrazolium dye from Amresco, USA. Briefly, we dissolved the tetrazolium salt in PBS at a concentration of 5 mg/ml and diluted 1 ml of this solution into 12 ml of DMEM culture media without FBS. Of this solution, we added 100 µl/well and incubated the plate at room temperature for 30 min. Note, metabolically active cells reduce the yellowish tetrazolium MTT substrate to a purple-colored insoluble formazan dye. We lysed the cells with 50 µL/well of dimethyl sulfoxide and monitored the absorbance at 570 nm in a multimode plate reader (EnSpire, PerkinElmer, USA). We analyzed the data by comparing DMSO vehicle-treated controls against the various drug treatments in Excel version 16.52. The data are %-cell viability and represent triplicate measurements of three independent experiments.

We treated the cell cultures as follows (Table 1): A: Single drug treatment at different concentrations with decitabine, 4-PBA or THU for up to 96 h and 48 h, respectively. B1: Daily combined treatment with THU at different concentrations and a fixed decitabine 3 µM concentration for 72 h. B2: Daily combined drug treatment of decitabine (3 µmolar) and 4-PBA (3 mmolar) for 72 h, followed by daily treatment of 3 mM 4-PBA for another 48 h. C1: Combined daily treatment of THU (40 µg/ml) and 3 µM decitabine for 72 h followed by daily treatment with THU (40 µg/ml) and 3 mM 4-PBA for another 48 h. C2: Gene knockdown of cytidine deaminase with the antisense oligonucleotide ASO-CDA for 24 h followed by the treatment schedule outlined in B2.

**Table 1:** Drug treatment schedule of Caco-2, HCT-116-p53 null and HCT-116-p53 wild-type cell cultures

Treatment regimen	Drug	Drug concentration	Treatment duration
Single drug treatment (A)	Decitabine (µM)	0.5, 1, 3, 6, 12, 25	24 h, 48 h, 72 h, 96 h
	4-PBA (mM)	0.5, 1, 3, 6, 12	24 h, 48 h, 72 h, 96 h
	THU (µg/ml)	1, 10, 20, 30, 40, 80	48 h
Gene knockdown of CDA	ASO-CDA (nM)	50	24 h, 48 h, 72 h, 96 h
Combined drug treatment (B1-B2)	THU & Decitabine (B1)	THU (1, 10, 20, 30, 40, 80 µg/ml) + 5-deoxycytidine (3 µM)	72 h
	Decitabine & 4-PBA (B2)	5-deoxycytidine (3 µM) + 4-PBA (3 mM) for 72 h, 4-PBA (3 mM) for another 48 h	120 h
Triple drug treatment (C1)	THU & Decitabine & 4-PBA	THU (40 µg/ml) + 5-deoxycytidine (3 µM) + 4-PBA (3 mM) for 72 h, THU (40 µg/ml) + 4-PBA (3 mM) for another 48 h	120 h
Gene knockdown of CDA and combined epigenetic drug treatment (C2)	ASO-CDA & Decitabine & 4-PBA	ASO-CDA (50 nM) 24 h, 5-deoxycytidine (3 µM) + 4-PBA (3 mM) for 72 h, 4-PBA (3 mM) for another 48 h	144 h

#### BrdU proliferation assay

We performed the 5-bromo-2-deoxyuridine (BrdU) labeling assay according to the manufacturer’s recommendations (Roche, Switzerland). We seeded 2 × 10^3^ cells of Caco-2, HCT-116-p53wt and HCT-116-p53null cells into 96-well plates and performed the assay in a final volume of 100 µL of culture media. After 24 h in culture, the drug treatment schedules and the gene knockdown experiments were identical to the ones described above (Table 1). After the various treatments, we added the BrdU labeling reagent for 2 h. Subsequently, we removed the labeling medium and fixed the cells with the reagent FixDenat for 30 min. Once again, we removed the reagent, added 100 µl of the anti-BrdU-POD working solution and incubated the samples for 90 min at room temperature. Thereafter, we removed the working solution, washed the cells repeatedly with PBS and added 100 µl of peroxidase substrate solution for an incubation period of 5–10 min. Finally, we measured the incorporation of BrdU into DNA at 450 nm in a multimode plate reader (EnSpire, PerkinElmer, USA).

#### EdU cell imaging assay

We seeded 1 × 10^5^ cells/well in a 12-well plate and allowed the cells to adhere for 8 h. The drug treatment consisted of decitabine (3 µM), 4-PBA (3 mM), and we evaluated the combined treatment for up to 120 h. We performed the EdU assay according to the manufacturer’s recommendations (Click-iT® Plus EdU Imaging Kits, Thermo Fisher, USA) and treated the cells with 10 µM EdU reagent at 37℃ for 2 h. Note that the modified thymidine analogue EdU (5-ethynyl-2′-deoxyuridine) is incorporated into DNA, and this nucleoside is labeled with an Alexa Fluor® dye that can be visualized by fluorescence microscopy. Subsequently, we fixed the cells with 3.7% paraformaldehyde for 15 min and removed the fixative by washing them with 3% BSA (Sigma-Aldrich, Germany). Next, we permeabilized the cells with 0.5% Triton X-100 for 20 min, followed by a washing step with PBS. Subsequently, we added 500 µl of the Click-iT® Plus cocktails to each well and allowed to react for 30 min. Finally, we treated the cells with 2 µg/ml of the nuclear counter stain Hoechst 33,342 for 15 min and examined the fluorescent cells with a Nikon Eclipse Ti Series microscope. We captured the data with the Nikon software NIS-Elements AR 5.02.03 64bit and counted the number of EDU and Hoechst 33,342 labeled cells.

### Cell cycle analysis by flow cytometry

We seeded 1 × 10^5^ cells/well on a six well plate and allowed the cells to adhere for 8 h. The drug treatment consisted of decitabine (3 µM), 4-PBA (3 mM), and we evaluated the combined treatment for up to 120 h. We harvested the cells by trypsinization using standard operating protocols (0.25% trypsin and 0.53 mM EDTA) and washed them with PBS. Subsequently, we stained the cells with 5 µM Vybrant® DyeCycle™ Violet (DCV) (Thermo Fisher, USA) and incubated them at 37℃ for 30 min. Finally, we performed the cell cycle analysis with a BD™ LSR II flow cytometer (BD, USA) by evaluating the fluorescence at ~ 405 nm excitation and ~ 440 nm emission. We analyzed the data with the FlowJo software version 10.4 (BD, USA).

### Western blot assay

We prepared cell lysates in a RIPA lysis and extraction buffer (Thermo Fisher, Germany) and inhibited proteases, phosphatases and deacetylase with the Thermo Scientific™ Halt™ Protease, Phosphatase Inhibitor Cocktail (100X) and Deacetylase Cocktail (100X) according to the manufacturers’ recommendations (Thermo Fisher and APExBIO, Germany). We determined the protein concentration of the cell lysates with the Pierce™ Detergent Compatible Bradford Assay Kit (Thermo Fisher, Germany), and separated 20 µg of total protein on 8–12% sodium dodecyl sulfate polyacrylamide gels (SDS-PAGE). We performed Western blotting with the Trans-Blot Turbo Transfer System (Bio-Rad, USA). To avoid unspecific binding, we incubated the membranes with 5% skim milk powder at room temperature for 1 h. WB experiments were performed with anti-DNMT1 (1:1000, CST, USA), anti-p53 (1:1000, Santa Cruz Biotechnology, USA), anti-JAK1 (1:1000, Santa Cruz, USA), Apoptosis antibody sample kit (1:1000, CST, USA), anti-PPAR r (1:1000, Santa Cruz, USA), anti-Acetyl-H3 sample kit (1:1000, CST, USA), anti-ß-Tubulin (1:1000, Sigma Aldrich, USA), anti-CDA (1:1000, CST, USA) antibodies overnight at 4 °C. Subsequently, the membranes were washed 3 × for 10 min each with a TBST buffer and incubated with either the anti-mouse IgG, HRP-linked antibody #7076 (CST) secondary antibodies or anti-rabbit IgG, HRP-linked antibody #7074 at room temperature for 1 h. To visualize the bands, we employed the Amersham ECL Prime kit (Cytiva, UK) and imaged the immunoblots on a ChemiDoc XRS + Imaging System (Bio-Rad, USA). We quantified the target proteins relative to ß-tubulin (housekeeping protein). All experiments were performed at least three times, and the data are given relative to the DMSO vehicle control.

### Gene silencing of CDA by s antisense LNA GapmeRs

We silenced the CDA with an antisense strategy termed LNA GapmeRs. The LNA GapmeRs function as single-stranded, antisense oligonucleotide and catalyze RNase H-dependent degradation of RNA targets (QIAGEN, Hilden, Germany). The sequence of the CDA GapmeR is 5′-3′ TAGGCTGGACTTTGAA, and the concentration of the GapmeR silencing probes was set to 50 nM. We used scrambled RNA (50 nM) as negative control for gene silencing experiments.

### Whole-genome gene expression and miRNA expression profiling

We previously reported the experimental procedures to perform whole-genome transcript profiling [39]. Briefly, we isolated total RNA from Caco-2 cells after decitabine (3 µM), 4-PBA (3 mM) and the combined treatment and followed the Affymetrix Gene Chip® Expression Analysis Technical Manual (Affymetrix, USA). We prepared cDNA followed by an in vitro transcription step to obtain copy RNA. Based on metal-induced hydrolysis we obtained fragmented cRNA, which we hybridized onto the Affymetrix Gene chip HG-U133 version 2.0 array (human). After scanning of the arrays, we normalized the signal intensity data with the robust multi-array average (RMA) algorithm of the Gene Expression Console software for background-adjusted and log-transformed perfectly matched individual probes. Subsequently, we uploaded the data onto the GeneXplain 3.0 platform (http://platform.genexplain.com/bioumlweb) and computed t-test for statistical analysis of differentially expressed genes (DEGs) by comparing DMSO vehicle controls against the various treatments. Only DEGs adjusted for the false discovery rate (FDR) p < 0.05, and fold change (FC) > 1.5 was considered for further analysis. Essentially, we performed the miRNA gene expression analysis as described above and more detailed in our previous publication [39].

### Functional enrichment analysis

We performed gene ontology (GO) enrichment analysis to explore the biological functions of DEGs. We queried the DAVID Bioinformatic Resources v6.8 (https://david.ncifcrf.gov/) and the Metascape (https://metascape.org/gp/index.html#/main/step1) database to identify enriched terms. We visualized the results as bar charts and p-values for significantly enriched terms.

### Chromosome analysis of drug response genes and miRNAs

We used the R version 4.0.2, R package *RIdeogram* (https://cran.r-project.org/web/packages/RIdeogram/index.html) and the *PhenoGram Plot* tools (http://visualization.ritchielab.org/phenograms/create) to map and visualize the chromosomal location of genes across the whole human genome. We downloaded the annotation files from the GENCODE website (https://www.gencodegenes.org/) and mapped the gene density information.

### Prediction of CpG islands and DNA methylation status

We considered the genomic data of 34 tumor-free peritumoral tissue sets, which served as controls and evaluated the DNA methylation status among 288 colon adenocarcinoma cases of the TCGA Pan-Cancer cohort. We focused on drug-responsive genes following decitabine and/or PBA treatment of the Caco-2 cell line and used the SMART online Shiny Methylation Analysis Resource Tool to define DNA methylation patterns (http://www.bioinfo-zs.com/smartapp/). We searched for CpG islands with the MethPrimer (http://www.urogene.org/cgi-bin/methprimer/methprimer.cgi) and DBCAT (http://dbcat.cgm.ntu.edu.tw/) software by interrogating promoter sequences of drug responsive genes 4,000 nucleotides upstream of its transcription start site. Statistical significance was calculated in Graphpad Prism 9 (Graphpad Software, USA).

### Survival analysis

We used the GEPIA 2 online tool (http://gepia2.cancer-pku.cn/#index) to explore the prognostic value of treatment-responsive genes following epigenetic drug treatment of colon cancer cell lines. In Kaplan–Meier plots, we determined the clinical relevance of drug-responsive genes for survival by dividing 270 colon cancer patients into low- and high-expression individuals based on the 50% median expression level as cutoff.

### Statistical analyses

The data are mean ± SD (standard deviation) of at least three independent experiments, and each experiment consisted of triplicate repeats. We processed the data in Prism GraphPad Prism 9 or the R software and depending on the data distribution used the Student’s T-test (cell cycle analysis, BrdU, EdU), the Mann–Whitney or Wilcoxon test and ANOVA (MTT assay). We considered a p-value < 0.05 as statistically significant.

## Supplementary Information

**Additional file 1. Table S1**. Inhibition of cell viability.

**Additional file 2. Fig. S1**. Cell cycle analysis and inhibition of cell proliferation.

**Additional file 3. Table S2**. Differentially expressed genes and miRNAs following drug treatment.

**Additional file 4. Table S3**. Induced expression of apoptosis-related genes.

**Additional file 5. Table S7**. Tumor suppressor miRNAs.

**Additional file 6. Table S4.1**. The chromosomal distribution of drug responsive DEGs.

**Additional file 7. Table S5.1**. CpG islands information in promoters of decitabine responsive genes. **Table S5.2**. CpG islands information in promoters of decitabine and PBA responsive genes.

**Additional file 8. Fig. S2**. Decitabine- and/or PBA-responsive genes across different chromosomes.

**Additional file 9. Table S6**. Kaplan-Meier survival estimates of epi-drug responsive genes in colon cancer patients.

**Additional file 10. Table S8**. Primer sequence for qPCR assays.

## Data Availability

All data generated or analyzed during this study are included in this published article and its Additional files.

## Abbreviations

5-Aza: 5-Aza-2′-deoxycytidine
5-Fu: 5-Fluorouracil
ATCC: American Type Culture Collection
BID: BH3-interacting domain death agonist
BRAF: B-raf proto-oncogene
BrdU: Bromodeoxyuridine
CCCL: Colon cancer cell line
CDA: Cytidine deaminase
CENP-A: Centromere protein A
CHK2: Checkpoint kinase 2
CRC: Colorectal cancer
DEGs: Differentially expressed genes
DNMTs: DNA methyltransferases
DUSP6: Dual specificity phosphatase 6
EdU: Ethynyldeoxyuridine
EGFR: Epidermal growth factor receptor
ER: Endoplasmic reticulum
FACS: Fluorescence-activated cell sorting
FAM162A: Family with sequence similarity 162 member A
FBS: Fetal bovine serum
FDA: Food and Drug Administration
FDR: False discovery rate
GEPIA2: Gene Expression Profiling Interactive Analysis 2
GO: Gene ontology
HDAC1: Histone deacetylase 1
HDACi: Histone deacetylase inhibitor
HR: Hazard ratio
JAK1: Janus kinase 1
LBR: Lamin B receptor
miRNA: MicroRNA
ncRNA: Non-coding RNA
NDRG1: N-Myc downstream regulated 1
NCOA3: Nuclear receptor coactivator 3
NCCN: National comprehensive cancer network
p53wt: P53 wild type
PARP: Poly (ADP-ribose) polymerase
PBA: Sodium phenylbutyrate
PCA: Principal component analysis
PERP: P53 apoptosis effector
PEPT1: Solute carrier family 15 member 1
PFS: Progression-free survival
RECIST: Response evaluation criteria in solid tumors
RIPA: Radio-immunoprecipitation assay
RT-qRCR: Quantitative reverse transcription PCR
SAFA/ HNRNPU: Heterogeneous nuclear ribonucleoprotein U
SAM: S-adenosylmethionine
SEMA6A: Semaphorin 6A
SHARP: Nuclear receptor corepressor 2/histone deacetylase 1 associated repressor protein
SLC15A1/ MAEL: Maelstrom spermatogenic transposon silencer
SMART: Shiny methylation analysis resource tool
TNFSF10/ TRAIL: Tumor necrosis factor (ligand) family, member 1
THU: Tetrahydrouridine
VEGF: Vascular endothelial growth factors
WHO: Word Health Organization
XCI: X-chromosome inactivation
XIST: X-inactive specific transcript
