# Supplementary figures and images for: Combined inhibition of histone deacetylase and cytidine deaminase improves epigenetic potency of decitabine in colorectal adenocarcinomas
Source: Clin Epigenetics. 2023 May 19;15:89. doi: 10.1186/s13148-023-01500-1 (PMC10199547; doi:10.1186/s13148-023-01500-1)

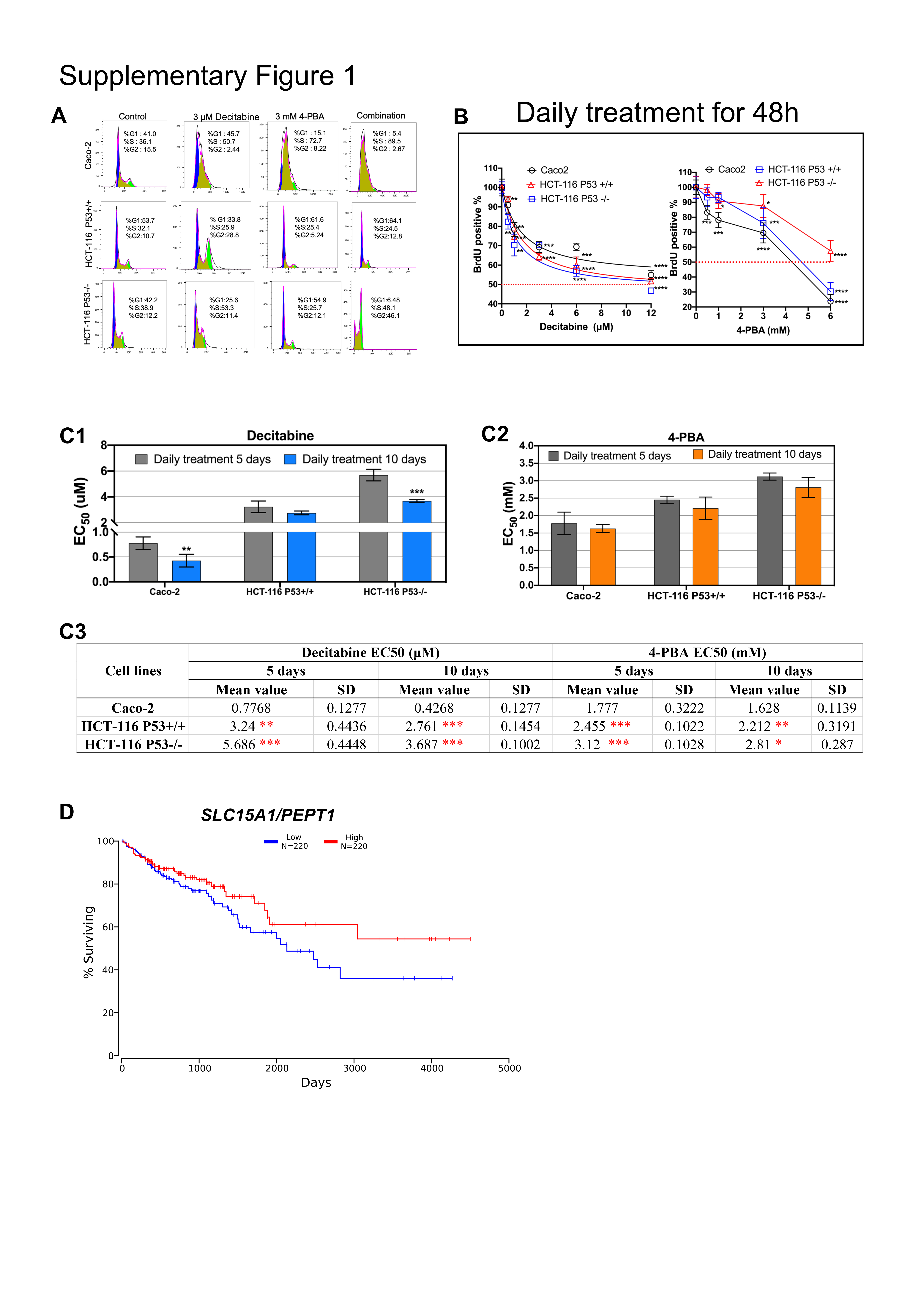

Supplement: Supplementary file 2 — Additional file 2. Fig. S1. Cell cycle analysis and inhibition of cell proliferation. [file 13148_2023_1500_MOESM2_ESM.jpg]

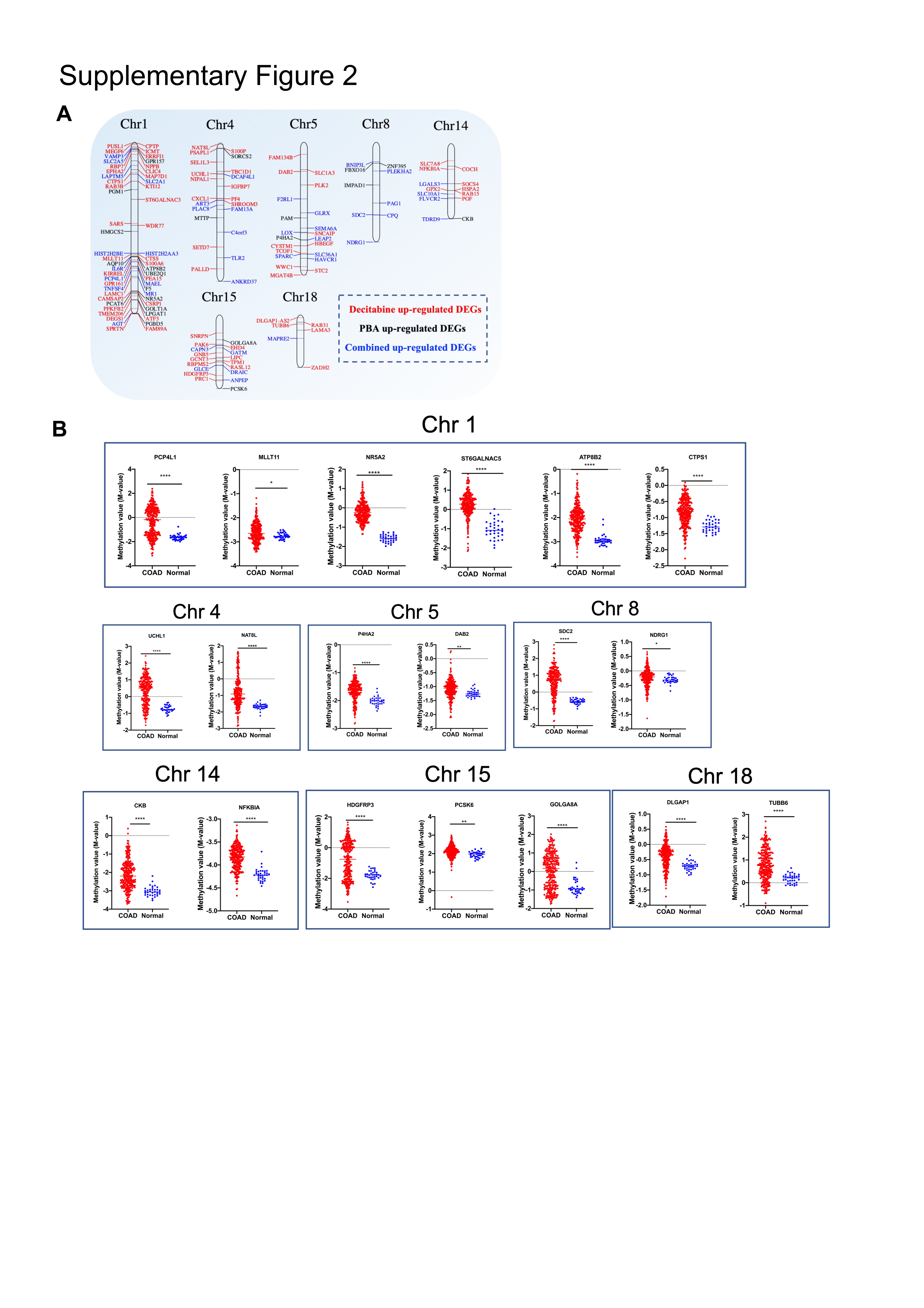

Supplement: Supplementary file 8 — Additional file 8. Fig. S2. Decitabine- and/or PBA-responsive genes across different chromosomes. [file 13148_2023_1500_MOESM8_ESM.jpg]
