# Supplementary material for: Combined inhibition of histone deacetylase and cytidine deaminase improves epigenetic potency of decitabine in colorectal adenocarcinomas
Source: Clin Epigenetics. 2023 May 19;15:89. doi: 10.1186/s13148-023-01500-1 (PMC10199547; doi:10.1186/s13148-023-01500-1)
Supplement: Supplementary file 5 — Additional file 5. Table S7. Tumor suppressor miRNAs. [file 13148_2023_1500_MOESM5_ESM.docx]

**Supplementary table7: Tumor suppressor miRNAs.**

| **Number** | **miRNA** | **Accession number** | **Chr** | **Fold change** | **FDR** | **Category** | **Function** | **reference** | **PMID** |
| --- | --- | --- | --- | --- | --- | --- | --- | --- | --- |
| 1 | hsa-miR-517a-3p | MIMAT0002852 | chr19 | 46.93 | 1.63E-07 | TSG | apoptosis | Restoration of miR-517a expression induces cell apoptosis in bladder cancer cell lines | PMID: 21479368 |
| 2 | hsa-miR-512-3p | MIMAT0002823 | chr19 | 34.54 | 0.0000014 | TSG | apoptosis | miR-512-3p acts as a cell-type-specific tumor suppressor | PMID: 32974790 |
| 3 | hsa-miR-516b-5p | MIMAT0002859 | chr19 | 28.81 | 0.00000117 | TSG | anti-proliferation | miR-516b functions as a tumor suppressor by directly modulating CCNG1 expression in esophageal squamous cell carcinoma | PMID: 30119241 |
| 4 | hsa-miR-525-5p | MIMAT0002838 | chr19 | 24.06 | 1.98E-07 | TSG | anti-proliferation/invasion | miR-525-5p negatively regulates cell proliferation, migration, invasion, and epithelial–mesenchymal transition in glioma, | PMID: 33116581 |
| 5 | hsa-miR-1323 | MIMAT0005795 | chr19 | 22.23 | 1.63E-07 | TSG | anti-migration | Low-miR-1323 levels were associated with lymph node metastasis and advanced clinical stage. | PMID: 32211853 |
| 6 | hsa-miR-519c-5p | MIMAT0002831 | chr19 | 21.88 | 2.41E-07 | TSG | anti-proliferation | miR‑519 overexpression and downregulation inhibited and stimulated MCF‑7 cell proliferation, | PMID: 31966082 |
| 7 | hsa-miR-526a | MIMAT0002845 | chr19 | 13.74 | 0.00000161 | TSG | anti-proliferation | MicroRNA-526a targets p21-activated kinase 7 to inhibit tumorigenesis in hepatocellular carcinoma | PMID: 28560394 |
| 8 | hsa-miR-520c-5p | MIMAT0005455 | chr19 | 13.74 | 0.00000161 | TSG epigenetic | anti-migration and invasion | miR-505-5p and miR-520c-3p target the 3′-UTR of S100A4 and inhibits its expression and its mediated migration and invasion. | PMID: 28423501 |
| 9 | hsa-miR-498 | MIMAT0002824 | chr19 | 11.68 | 0.00000859 | TSG | anti-proliferation | MiR-498 regulated FOXO3 expression and inhibited the proliferation of human ovarian cancer cells | PMID: 26054675 |
| 10 | hsa-miR-526b-5p | MIMAT0002835 | chr19 | 10.71 | 0.0000145 | TSG | anti-invasion | miR-526b suppressed epithelial-to-mesenchymal transition (EMT) of HCC cells. | PMID: 29152116 |
| 11 | hsa-miR-520a-5p | MIMAT0002833 | chr19 | 4.76 | 0.0000114 | TSG | anti-proliferation, invasion | Long noncoding RNA SOX21-AS1 regulates the progression of triple-negative breast cancer through regulation of miR-520a-5p/ORMDL3 axis | PMID: 32277517 |
| 12 | hsa-miR-515-3p | MIMAT0002827 | chr19 | 4.6 | 0.0000191 | TSG | anti-invasion, migration | miR-515-3p could decrease the expression of the mesenchymal markers, and more importantly, suppress invasion and metastasis of ESCC cells. | PMID: 33243974 |
| 13 | hsa-miR-520d-3p | MIMAT0002856 | chr19 | 4.59 | 0.0006 | TSG | anti-proliferation | MiR-520d-3p antitumor activity in human breast cancer via post-transcriptional regulation of spindle and kinetochore associated 2 expressions. | PMID: 29736203 |
| 14 | hsa-mir-525 | MI0003152 | chr19 | 4.58 | 0.0001 | TSG | anti-proliferation, invasion | miR-525-5p negatively regulates cell proliferation, migration, invasion, and epithelial–mesenchymal transition in glioma, | PMID: 33116581 |
| 15 | hsa-miR-518b | MIMAT0002844 | chr19 | 4.56 | 0.0001 | TSG | anti-proliferation | MicroRNA-518b functions as a tumor suppressor in glioblastoma by targeting PDGFRB | PMID: 28849154 |
| 16 | hsa-mir-519b | MI0003151 | chr19 | 4.54 | 0.0000338 | TSG | anti-proliferation, invasion | MiR-519b-3p Inhibits the Proliferation and Invasion in Colorectal Cancer via Modulating the uMtCK/Wnt Signaling Pathway | PMID: 31312141 |
| 17 | hsa-miR-520c-3p | MIMAT0002846 | chr19 | 4.3 | 0.0000208 | TSG | anti-proliferation | MicroRNA-520c-3p functions as a novel tumor suppressor in lung adenocarcinoma | PMID: 30942957 |
| 18 | hsa-miR-130a-3p | MIMAT0000425 | chr11 | 3.86 | 2.37E-05 | TSG | apoptosis | MiR-130a-3p | PMID: 28487475 |
| 19 | hsa-miR-512-5p | MIMAT0002822 | chr19 | 3.18 | 0.0002 | TSG | apoptosis | miR-512-5p suppresses proliferation, migration and invasion, and induces apoptosis in non-small cell lung cancer cells by targeting ETS1 | PMID: 30896817 |
| 20 | hsa-miR-6768-5p | MIMAT0027436 | chr16 | 3.13 | 0.0012 | TSG | apoptosis | Circular RNA hsa_circ_0000848 Promotes Trophoblast Cell Migration and Invasion and Inhibits Cell Apoptosis by Sponging hsa-miR-6768-5p | PMID: 32509771 |
| 21 | hsa-mir-516b-2 | MI0003167 | chr19 | 2.67 | 0.0001 | TSG | anti-proliferation | miR-516b functions as a tumor suppressor by directly modulating CCNG1 expression in esophageal squamous cell carcinoma | PMID: 30119241 |
| 22 | hsa-miR-184 | MIMAT0000454 | chr15 | 2.51 | 0.0129 | TSG | apoptosis | Tumor Suppressor miR-184 Enhances Chemosensitivity by Directly Inhibiting SLC7A5 in Retinoblastoma | PMID: 31803607 |
| 23 | hsa-miR-3622a-5p | MIMAT0018003 | chr8 | 2.52 | 0.0169 | TSG | apoptosis | Tumor suppressor role of miR-3622b-5p in ERBB2-positive cancer | PMID: 28160563 |
