# Supplementary material for: Combined inhibition of histone deacetylase and cytidine deaminase improves epigenetic potency of decitabine in colorectal adenocarcinomas
Source: Clin Epigenetics. 2023 May 19;15:89. doi: 10.1186/s13148-023-01500-1 (PMC10199547; doi:10.1186/s13148-023-01500-1)
Supplement: Supplementary file 9 — Additional file 9. Table S6. Kaplan-Meier survival estimates of epi-drug responsive genes in colon cancer patients. [file 13148_2023_1500_MOESM9_ESM.docx]

**Supplementary Table 6. Kaplan-Meier survival of epi-drug responsive genes in colon cancer patients.**

| **Epi-Drug** | **Gene** | **GEPIA2** | | **Regulations** | |
| --- | --- | --- | --- | --- | --- |
|  |  | **Hazard ratio** | **Log-rank p- value** | **Fold change** | **FDR** |
| **Decitabine** | NOX1 | 0.63 | 0.05 | 2.03 | 0.0115 |
|  | LPCAT3 | 0.61 | 0.048 | 1.75 | 0.0241 |
|  | ERI1 | 0.52 | 0.012 | 1.79 | 0.0109 |
|  | CTPS1 | 0.59 | 0.032 | 2.08 | 0.0176 |
|  | ADORA2B | 0.61 | 0.047 | 2.02 | 0.033 |
|  | ZNF33A | 2 | 0.0064 | -1.51 | 0.0291 |
|  | FADS1 | 1.7 | 0.031 | -1.6 | 0.0383 |
|  | PKP4 | 1.6 | 0.048 | -1.62 | 0.0473 |
|  | ANKRD36 | 2 | 0.0062 | -1.84 | 0.0341 |
|  | GMDS-AS1 | 1.7 | 0.039 | -1.67 | 0.0143 |
|  | RASA4 | 1.9 | 0.011 | -3.11 | 0.0052 |
|  | MGEA5 | 1.8 | 0.013 | -1.53 | 0.0281 |
| **PBA** | ZG16 | 0.61 | 0.044 | 4.41 | 0.0069 |
|  | HMGCS2 | 0.55 | 0.016 | 3.76 | 0.0392 |
|  | FBXO16 | 0.55 | 0.017 | 1.78 | 0.0367 |
|  | F2RL1 | 0.54 | 0.014 | 1.51 | 0.0398 |
|  | CACNA1G | 0.59 | 0.05 | 1.62 | 0.0259 |
|  | ARL17A | 1.7 | 0.04 | -2.21 | 0.0009 |
|  | BST2 | 1.8 | 0.018 | -2.58 | 0.002 |
| **Combined** | ABHD6 | 0.61 | 0.05 | 2.07 | 0.0234 |
|  | ZG16 | 0.61 | 0.044 | 2.91 | 0.0154 |
|  | ARL17A | 1.7 | 0.04 | -2.49 | 0.0007 |
|  | ANKRD13D | 1.7 | 0.039 | -1.66 | 0.0464 |
|  | BST2 | 1.8 | 0.018 | -2.53 | 0.0029 |
|  | LRRC37A2 | 1.9 | 0.011 | -1.69 | 0.0013 |
|  | STAG3L1 | 1.8 | 0.021 | -1.52 | 0.0192 |
