# Supplementary material for: Combined inhibition of histone deacetylase and cytidine deaminase improves epigenetic potency of decitabine in colorectal adenocarcinomas
Source: Clin Epigenetics. 2023 May 19;15:89. doi: 10.1186/s13148-023-01500-1 (PMC10199547; doi:10.1186/s13148-023-01500-1)
Supplement: Supplementary file 10 — Additional file 10. Table S8. Primer sequence for qPCR assays. [file 13148_2023_1500_MOESM10_ESM.docx]

**Supplementary Table 8. Primer sequence for qPCR assays**

| **Species** | **Gene** | **Sequences** |
| --- | --- | --- |
| Homo sapiens | CDA | L-5'-TGAAGCCTGAGTGTGTCCAG-3' |
|  |  | R-5'-TAGCAATTGCCCTGAAATCC-3' |
|  | DNMT1 | L-5'-GAGCTACCACGCAGACATCA-3' |
|  |  | R-5'-CGAGGAAGTAGAAGCGGTTG-3' |
|  | GAPDH | L-5'-TTCTCTGATTTGGTCGTATTGG-3' |
|  |  | R-5'-GTAGTTGAGGTCAATGAAGGG-3' |
|  | β-actin | L-5'-GGCATTGTTACCAACTGGGACG-3' |
|  |  | R-5'-CTCTTTGATGTCACGCACGATTTC-3' |
